# Procollagen C-Proteinase Enhancer-1 (PCPE-1) deficiency in mice reduces liver fibrosis but not NASH progression

**DOI:** 10.1371/journal.pone.0263828

**Published:** 2022-02-11

**Authors:** Patricia Sansilvestri Morel, Valerie Duvivier, Florence Bertin, Nicolas Provost, Adel Hammoutene, Edwige-Ludiwyne Hubert, Arantxa Gonzalez, Isabelle Tupinon-Mathieu, Valerie Paradis, Philippe Delerive

**Affiliations:** 1 Cardiovascular and Metabolic Diseases Research, Institut de Recherches Servier, Suresnes, France; 2 Pathology Department, Hôpital Beaujon, Paris, France; 3 Program of Cardiovascular Diseases, CIMA Universidad de Navarra, IdiSNA and CIBERCV, Pamplona, Spain; 4 Cardiovascular and Metabolic Diseases, Institut de Recherches Internationales Servier, Suresnes, France; University of Navarra School of Medicine and Center for Applied Medical Research (CIMA), SPAIN

## Abstract

**Background and aims:**

Nonalcoholic Steatohepatitis (NASH) is a major cause of end-stage liver diseases such as cirrhosis and hepatocellular carcinoma resulting ultimately in increased liver-related mortality. Fibrosis is the main driver of mortality in NASH. Procollagen C-Proteinase Enhancer-1 (PCPE-1) plays a key role in procollagen maturation and collagen fibril formation. To assess its role in liver fibrosis and NASH progression, knock-out mice were evaluated in a dietary NASH model.

**Methods:**

Global constitutive *Pcolce*^*-/-*^ and WT male mice were fed with a Choline Deficient Amino acid defined High Fat Diet (CDA HFD) for 8 weeks. Liver triglycerides, steatosis, inflammation and fibrosis were assessed at histological, biochemical and gene expression levels. In addition, human liver samples from control and NASH patients were used to evaluate the expression of PCPE-1 at both mRNA and protein levels.

**Results:**

*Pcolce* gene deficiency prevented diet-induced liver enlargement but not liver dysfunction. Furthermore, liver triglycerides, steatosis and inflammation were not modified in *Pcolce*^*-/-*^ male mice compared to WT under CDA HFD. However, a significant decrease in liver fibrosis was observed in *Pcolce*^*-/-*^ mice compared to WT under NASH diet, associated with a decrease in total and insoluble collagen content without any significant modifications in the expression of genes involved in fibrosis and extracellular matrix remodeling. Finally, PCPE-1 protein expression was increased in cirrhotic liver samples from both NASH and Hepatitis C patients.

**Conclusions:**

*Pcolce* deficiency limits fibrosis but not NASH progression in CDA HFD fed mice.

## Introduction

Nonalcoholic fatty liver disease (NAFLD) is a common and progressive disease mainly characterized by hepatic fat accumulation in the absence of alcohol consumption. NAFLD is strongly associated with obesity, metabolic syndrome, Type 2 Diabetes and dyslipidemia. NAFLD is subdivided into nonalcoholic fatty liver (NAFL) and nonalcoholic steatohepatitis (NASH) based on histological examination of liver biopsy and defined by the presence of inflammation and hepatocyte ballooning with various degrees of fibrosis [1, 2]. NAFLD is the most common cause of chronic liver disease worldwide with an estimated prevalence of 25%. In contrast to NAFL which is considered as a benign and reversible disease state, NASH accounts for an increased number of patients with cirrhosis, liver failure and hepatocellular carcinoma. NASH patients display an increased mortality compared to healthy population with a high cardiovascular risk. To date, there is no approved treatment for NASH. Long term follow-up studies revealed that fibrosis is the main driver of mortality in NASH [3, 4]. Fibrosis results from an excessive production of extracellular matrix (ECM) which is not balanced by degradation. From a mechanistic standpoint, it is believed that the accumulation of both triglycerides and pro-inflammatory and cytotoxic lipid oxidation side-products results in the formation of a necro-inflammatory milieu which triggers the activation of the main fibrogenic hepatic cell population, namely hepatic stellate cells (HSCs) [5]. HSCs are responsible for the deposition of the type I collagen-rich ECM and are key players in the development of NASH complications such as portal hypertension [6]. The ECM is a complex network of proteins including fibrillar and non-fibrillar collagens, glycosaminoglycans, proteoglycans and non-collagenous glycoproteins. In addition, this so-called matrisome composition may change with liver injury [7]. For instance, cross-linking of collagens makes ECM more resistant to degradation. Indeed, aging has been shown to enhance liver fibrotic response in mice through the impairment of extracellular matrix remodeling [8]. All of these studies underline the importance of better characterizing the matrisome and its remodeling during disease progression in order to identify potential drug targets.

PCPE-1(Procollagen C-Proteinase Enhancer-1, encoded by *PCOLCE* gene) has been described as an enhancer of BMP-1 (Bone Morphogenetic Protein-1, also named Procollagen C-Proteinase) [9], involved in the extracellular maturation of fibrillar procollagens. BMP-1 cleaves the C-terminal propeptides of fibrillar procollagens and this cleavage is a rate-limiting step in fibrogenesis, as monomers that retain C-terminal propeptides (unlike those retaining N-terminal propeptides) are not incorporated into fibrils [10]. PCPE-1 binds to C-terminal propeptides of fibrillar collagens (PICP (Procollagen type I C-terminal Propeptide) for instance) *via* its two CUB domains resulting in a conformational change that enhances BMP-1 activity [11, 12]. A homolog of PCPE-1, namely PCPE-2 (encoded by *PCOLCE2* gene) has been described with structural similarities of CUB domains [13]. PCPE-2 also has the ability to increase the C-terminal processing of procollagen by BMP-1 [13] suggesting a role in procollagen maturation but other physiological activities have been described such as a role in cholesterol transport [14]. To date, the *in vivo* role of PCPE-2 still needs to be elucidated.

Several studies have described a key role of PCPE-1 in the regulation of fibrosis in different organs such as heart [15], kidney [16], lung [17] or cornea [18] injury (for a review, see [19]). Little is known about the role of PCPE-1 in the development of liver fibrosis. While PCPE-1 is not detected in freshly-isolated rat hepatocytes, liver endothelial cells or Kupffer cells, it is poorly expressed by HSCs under basal condition but strongly induced by TGF-β (Transforming Growth Factor-β) in parallel to procollagen type I synthesis [20]. Moreover, PCPE-1 expression is increased in fibrotic livers of rats treated with CCl_4_ [20] suggesting that it may play a role in procollagen maturation during liver fibrogenesis. Hassoun *et al*. [21] have shown that plasma PCPE-1 levels in mice treated with CCl_4_ increased gradually during the progression of liver fibrosis and reflected the severity of the disease. Indeed, circulating PCPE-1 is the marker that is most well-correlated with fibrosis in animals administered fibrogenic chemicals [22]. This increased level of plasma PCPE-1 was also found in patients with liver fibrosis (Hepatitis C (HCV) or B virus patients) [23, 24]. Taken together, these results suggest that PCPE-1 could be a potential fibrosis biomarker.

This study explores the role of PCPE-1 in the development of NASH and liver fibrosis. Constitutive *Pcolce* knock-out mice were used in the CDA HFD (Choline Deficient Amino acid defined High Fat Diet)-induced NASH model. Typical parameters of NASH (liver steatosis, inflammation, fibrosis) were evaluated and compared to control mice. Finally, PCPE-1 mRNA and protein expression levels were assessed in cirrhotic liver specimens of NASH or HCV patients.

## Material and methods

### Animals

Mice were maintained on a 12:12 h light/dark cycle at 21 ± 2°C and had ad libitum access to tap water and standard or NASH diet. All procedures were performed according to the ethical protocol that has been approved by the Servier Institutional Animal Care and Use Committee in accordance with the French regulations (Decree n° 2013–118 from 01 February 2013 relative to the protection of animals used for scientific purposes and 4 orders of 01 February 2013).

Wild-type C57BL/6N and *Pcolce*^-/-^ mice (8 weeks-old, male and female) were obtained from the Transgenic Department of Charles River Laboratories (France). Generation of the *Pcolce*^-/-^ mice was performed by GenOway (Lyon, France) as previously described [25].

### NASH models

STAM^®^ model [26]: STAM liver samples were obtained from SMC Laboratories Inc. (Japan). To induce NASH, C57Bl/6J male mice were injected with streptozotocin (Sigma-Aldrich, 200 μg, subcutaneous) 2 days after birth. After weaning, mice were fed with a high fat diet (57% kcal of fat) from 4 week-old to 9 week-old. Control mice were injected with streptozotocin but were fed a standard diet from 4 to 9 week-old.

GAN DIO model (Gubra Amylin NASH Diet Induced Obese) [27]: liver samples were obtained from Gubra. (Denmark). To induce NASH, C57Bl/6J male mice were fed with standard diet or NASH-inducing diet which contains 40% saturated fat, 2% cholesterol, 22% fructose for 39 weeks.

CDA HFD (Choline Deficient Amino acid defined (0.1% methionine) High Fat Diet) model [28]: Upon arrival, male and female mice were randomly assigned to either control diet (A04 diet, SAFE) or NASH-inducing diet (A06071302, Research Diet) for 8 weeks. Mice were checked daily for health status and weighed once a week.

Western Diet model [29]: C57Bl/6J male mice (5-week-old) were purchased at Charles River Laboratories (France). Upon arrival, mice were assigned either to control diet (A04 diet, SAFE) or a NASH-inducing diet (D09100301, Research Diets), which contains 40% kcal of fat, 2% cholesterol and 20% fructose for 16 weeks.

For CDA HFD and Western Diet models, mice were anesthetized by isoflurane and livers were collected for gene expression analyses. For the CDA HFD model, blood samples were obtained from the heart cavity and livers were quickly removed, weighed and processed for histological and biochemical analyses.

### Gene expression studies

Total RNA was extracted using Qiagen RNA extraction kits following manufacturer’s instructions. Total RNA was treated with DNase I (Ambion Inc., Texas, USA) at 37°C for 30 minutes, followed by inactivation at 75°C for 5 minutes. Real time quantitative PCR (RT-qPCR) assays were performed using an Applied Biosystems 7500 sequence detector. Total RNA (1 μg) was reverse transcribed with random hexamers using High-Capacity cDNA Reverse Transcription Kit with RNase Inhibitor (Applied Biosystems, ThermoFisher Scientific) following the manufacturer’s protocol. Gene expression levels were determined by TaqMan Fast Universal PCR Master Mix (2x), No AmpErase UNG (ref:4352042, Applied Biosystem) and 18S (Hs99999901_S1, Applied Biosystems) transcript was used as an internal control to normalize the variations for RNA amounts. No difference in 18S expression was observed between the groups (regardless of genotype or diet). Gene expression levels are expressed relative to 18S mRNA levels. The following Taqman assays were used: *PCOLCE* qHsaCIP0027739 (Biorad); *PCOLCE2* qHsaCIP0031859 (Biorad); *Pcolce* qMmuCEP0056460 (Biorad); *Pcolce2* qMmuCEP0052963 (Biorad); *Bmp1* qMmuCEP0053968 (Biorad); *Acaca* Mm01304277_m1 (Applied Biosystems™); *Fasn* Mm00662319_m1 (Applied Biosystems™); *Srebf1* Mm00550338_m1 (Applied Biosystems™); *CD68* Mm00839636_g1 (Applied Biosystems™); *Il1b* Mm00434228_m1 (Applied Biosystems™); *TNF* Mm00443258_m1 (Applied Biosystems™); *Acta2* Mm02546133_m1 (Applied Biosystems™); *Col1a1* Mm00801666_g1 (Applied Biosystems™); *Loxl2* Mm00804740_m1 (Applied Biosystems™).

### Alanine AminoTransferase (ALT) and Aspartate Amino-Transferase (AST) analyses

Plasma levels of ALT and AST were determined with an automatic biochemical analyzer (Indiko Clinical Chemistry Analyzer, Thermofisher).

### Liver triglyceride content

Liver samples were processed for hepatic triglyceride (TG) content. Livers were homogenized and TG content was determined using an automatic biochemical analyzer (Indiko Clinical Chemistry Analyzer) with a Triglyceride assay kit (Thermofisher).

### Liver histology

Formalin-fixed, paraffin-embedded livers were sliced into 3-μm sections. Hematoxylin and Eosin (H&E) staining was performed to investigate liver histology and Picrosirius Red staining was used for liver fibrosis. NAFLD Activity Score (NAS) and fibrosis stage were determined by two double-blinded persons using the NASH CRN scoring system [30]. For hepatocellular steatosis, livers were classified into scores 0 to 3 (0: <5% of hepatocytes presenting steatosis, 1: 5 to 33% of hepatocytes presenting steatosis, 2: 34 to 66% of hepatocytes presenting steatosis and 3: > 67% of hepatocytes presenting steatosis). For inflammation, livers were scored into grades 0 to 3 (0: non inflammatory foci, 1: 1 inflammatory focus, 2: 2 to 4 inflammatory foci, 3: >4 inflammatory foci). Fibrosis was scored into stages from 0 to 4 (0: no fibrosis, 1: perisinusoidal or periportal fibrosis, 2: perisinusoidal and periportal fibrosis, 3: bridging fibrosis or septa, 4: cirrhosis).

### Liver collagen quantification

Livers were collected, dried on a paper towel, carefully cut into small pieces with a scalpel and snap-frozen into liquid nitrogen before storage at -80°C. Collagen was quantified according to Baicu *et al*. [31]. About 80 mg of wet liver tissue were sampled and dried at 60°C. Dry tissue was ground in a glass Potter and powder was weighed. Collagen was hydrolyzed with HCl 6N (50 mg dry tissue/mL) at 110°C overnight and evaporated at 80°C for 48h. Pellets were diluted in H_2_O (50 mg dry tissue/mL) and agitated for 2h at room temperature. Samples were then centrifuged 5 min at 450 g and supernatants were collected. OH-proline from tissues (and OH-proline standard (Sigma)) was then quantified by oxidation with 1 volume of chloramine T solution (0.14% (w/v) chloramine T (Sigma), 30% (v/v) ethylene glycol monomethyl ether, 50% (v/v) citrate buffer pH6) and incubated for 20 min at room temperature. Reaction was stopped by addition of 1 volume of perchloric acid 3.15M, and 1 volume of Ehrlich reagent (20% (w/v) 4-dimethylamino benzaldehyde (Sigma) in ethylene glycol) was added for 20 min at 60°C under agitation. Absorbance was then read in a spectrophotometer at 557nm.

For insoluble collagen: Tissue was diluted in NaCl 1N with proteases inhibitors (Complete Mini (Roche)) with a concentration of 50 mg dry tissue/ mL and incubated overnight at 4°C under agitation. Samples were centrifuged at 250 g for 5 min. Pellets were collected (mainly insoluble collagen) and evaporated at 80°C overnight. Hydrolyzation of insoluble collagen and OH-proline quantification were then performed as described above.

Quantity of collagen/ dry tissue weight was calculated by interpolation from OH-proline standards x 7.46 [32].

### Human liver samples

Liver tissues were obtained either from patients undergoing liver resection at the digestive surgical department of Beaujon Hospital (Clichy, France) for primary or secondary liver cancer, or from the explanted liver of patients undergoing liver transplantation. Patients’ clinical information is presented in Tables 1 and 2. All patients gave a written consent to participate in the study. The study was approved by the Institutional Review Board HUPNVS, University of Paris, AP-HP (IRB n° 00006477 and declaration n° DC-2009-936). The study conformed to the ethical guidelines of the 1975 Declaration of Helsinki. Liver specimens were examined by a pathologist and samples were taken from the most distal non tumoral tissue surrounding the tumoral part (for livers obtained from patients undergoing liver resection for primary or secondary liver cancer). Cirrhotic samples were obtained either from patients with metabolic syndrome or from patients with hepatitis C virus. Samples that had no abnormalities at liver histological examination were used as controls (hereafter referred as healthy liver).

**Table 1:** Patient’s characteristics. Liver samples used for RT qPCR analysis.

	Patient	Gender^a^	SAF score^b^
**Control**	1	M	S0A0F0
	2	M	S0A0F0
	3	M	S0A0F0
	4	M	S0A0F0
	5	F	S0A0F0
	6	F	S0A0F0
	7	M	S0A0F0
	8	M	S0A0F0
**NASH**	9	M	S2A3F4
	10	M	S2A4F3
	11	M	S1A4F4
	12	M	S1A3F3
	13	M	S1A3F4

^a^M: Male, F: Female.

^b^SAF score: Steatosis, Activity and Fibrosis score.

**Table 2:** Patient’s characteristics. Liver samples used for Western blot analysis.

	Patient	Gender^b^	SAF score^c^	METAVIR score
**Healthy liver**	1	F	S0A0F0	A0F0
	2	F	S0A0F0	A0F0
	3	M	S0A0F0	A0F0
	4	M	S0A0F0	A0F0
**NASH**	5	F	S1A3F4	-
	6	M	S2A3F4	-
	7	M	S2A3F4	-
	8	M	S0A1F4	-
**HCV**^**a**^ **related cirrhosis**	9	M	-	A1F4
	10	F	-	A0F4
	11	M	-	A1F4
	12	M	-	A0F4

^a^HCV: hepatitis C virus.

^b^F: female; M: male.

^c^SAF score: Steatosis, Activity and Fibrosis score.

### Western blotting analysis of human liver samples

About 50 mg of snap frozen human tissues were homogenized in RIPA buffer containing 150 mmol/L NaCl, 50 mmol/L TrisHCl, pH 7.4, 2 mmol/L EDTA, 0.5% sodium deoxycholate, 0.2% sodium dodecyl sulfate, 2 mmol/L activated sodium orthovanadate, complete protease inhibitor cocktail tablet (Complete mini, Roche) and complete phosphatase inhibitor cocktail tablet (PhosSTOP™, Roche). Lysates were centrifuged (12,000 g, 10 min, 4°C), supernatants were collected, and protein content was quantified using the Lowry protein assay (DC™ Protein Assay, Bio-Rad). Lysates were mixed with the reducing sample buffer for electrophoresis and subsequently transferred onto nitrocellulose membrane (Bio-Rad). Equal loading was checked using Ponceau red solution. Membranes were incubated with primary antibodies (Rat anti-Human PCPE-1 antibody, R&D systems MAB2627, 1/500; Mouse anti-GAPDH antibody, Millipore MAB374, 1/20,000). After secondary antibody, immunodetection was performed using an enhanced chemiluminescence kit (Immun-Star™ WesternC™ kit, Bio-Rad). Bands were revealed using the ChemiDoc imaging system (Bio-Rad). Values reported from Western blots were obtained by band density analysis using Image Lab software (Bio-Rad) and expressed as the ratio of PCPE-1/ GAPDH. Three independent experiments were done (3 gels), and quantification was averaged for the final representation.

### Statistical analysis

For comparison of 2 groups, an unpaired Student *t* test was used (GraphPad Prism® software, v9.0) after verification of the normal distribution of data. For more than 2 groups, a one-way analysis of variance was performed followed by a Tukey’s test. For body weight, a two-way analysis of variance was performed followed by a Tukey’s test. For the NAS histological parameters, a one-way analysis of variance with group (2 strains and 2 diets combined) as fixed effect was performed, followed a Tukey’s test (SAS software, v9.4). For the other histological parameters (scores of steatosis, inflammation and fibrosis), a Fisher exact test was performed to compare groups. Significance threshold was 5%.

## Results

### Liver PCPE-1 expression in different murine models of NASH

**Fig 1:**
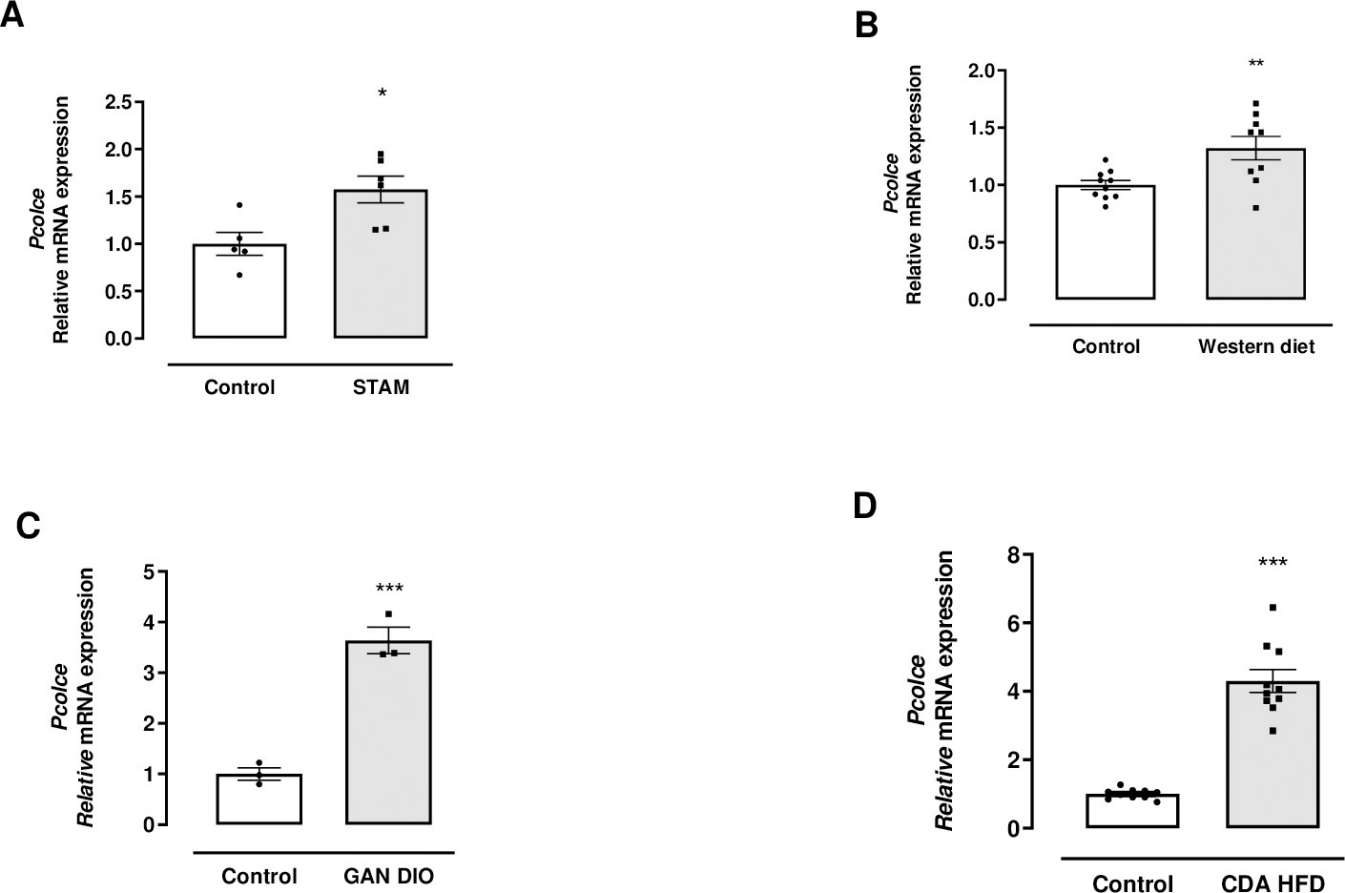
Evaluation of liver *Pcolce* mRNA gene expression in murine models of NASH. A: STAM^TM^ model, control mice (n = 5), STAM mice (n = 6); B: Western diet model, control (n = 10), Western diet mice (n = 9); C: GAN DIO model (n = 3/group); D: CDA HFD after 8 weeks of diet (n = 10/group). Data are expressed as mean ± SEM. *p<0.05, **p<0.01, ***p<0.001 *vs*. control mice. Unpaired *t* test.

In order to address the role of PCPE-1 in the development of NASH and liver fibrosis, we first evaluated its expression in different preclinical murine models of NASH. As shown in Fig 1, liver *Pcolce* mRNA expression was significantly increased in STAM^TM^ (x1.5), Western Diet (x1.3), GAN DIO (x3.6) and CDA HFD (x4.3) models. Interestingly, PCPE-1 up-regulation was more pronounced in mice models with established fibrosis such as GAN DIO and CDA HFD models [33].

### Liver mRNA expression of *Pcolce*, *Pcolce2* and *Bmp1* in *Pcolce*^-/-^ male mice

To evaluate the impact of PCPE-1 deficiency on typical NASH-associated parameters (liver steatosis, inflammation and fibrosis), the CDA HFD (8 weeks of diet) model was selected and investigated in WT and *Pcolce*^-/-^ male and female mice.

Constitutive *Pcolce*^-/-^ mice were generated as previously described [25]. Extensive phenotyping revealed that *Pcolce* KO mice do not display any gross abnormalities under basal conditions [25, 34]. Confirmation of *Pcolce* knock-out in the liver was assessed by RT-qPCR (S1 Fig). Expression of liver *Pcolce2*, its close homolog, was slightly but significantly (p<0.05) decreased whereas liver *Bmp1* mRNA expression was not modified in *Pcolce*^-/-^ compared to WT male mice (S1 Fig).

### Evaluation of body and liver weight, liver triglyceride, plasma ALT and AST levels in WT and *Pcolce*^-/-^ male mice under CDA HFD

**Fig 2:**
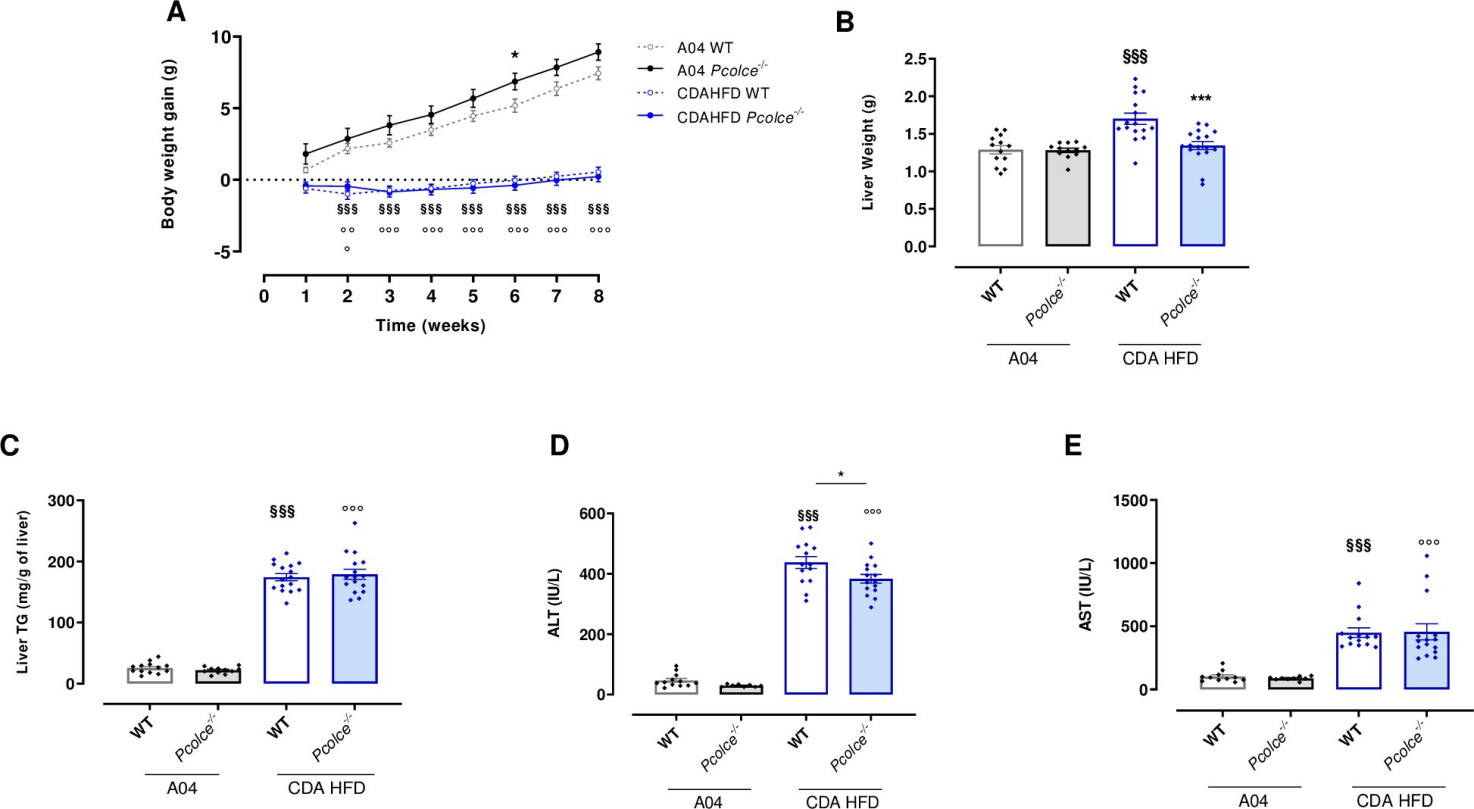
Body and liver weight, liver TG, ALT and AST levels in WT and *Pcolce*^-/-^ male mice under A04 or CDA HFD after 8 weeks. A: Body weight gain during A04 or CDA HFD diet (n = 12–18). ^§§§^p<0.001 WT A04 *vs*. WT CDA HFD; °°°p<0.001 *Pcolce*^-/-^ A04 *vs*. *Pcolce*^-/-^ CDA HFD; *p<0.05 WT A04 *vs*. *Pcolce*^-/-^ A04, Two-way ANOVA with Tukey’s post-hoc analysis. B: Liver weight (n = 12–18); C: Liver triglyceride (TG) content (n = 11–16); D: Plasma Alanine Aminotransferase (ALT) level (n = 8–15); E: Plasma Aspartate Aminotransferase (AST) level (n = 8–15). Panels B-E: ^§§§^p<0.001 *vs*. WT A04; °°°p<0.001 *vs*. *Pcolce*^-/-^ A04; *p<0.05 *vs*. WT CDA HFD. One-way ANOVA with Tukey’s post hoc analysis. Data are expressed as mean ± SEM.

To evaluate the impact of PCPE-1 deficiency on NASH progression and development of fibrosis, WT and *Pcolce*^-/-^ male mice were subjected to CDA-HFD for 8 weeks. As shown in Fig 2A, no body weight gain was observed during the 8 week-NASH diet for both WT and *Pcolce*^-/-^ male mice. A slightly higher body weight gain was observed in *Pcolce*^-/-^ male mice compared to male WT under control diet (Fig 2A).

Liver weight was significantly increased in WT male mice under CDA HFD compared to control diet (Fig 2B). *Pcolce* gene deficiency completely prevented this increase in liver mass (Fig 2B).

The content of liver triglyceride (TG) was similarly increased in both WT and *Pcolce*^-/-^ mice under CDA HFD (Fig 2C).

CDA HFD induced a large increase in plasma ALT levels in both WT mice and *Pcolce*^-/-^ mice, but to a lesser extent in PCPE-1 deficient mice (Fig 2D). By contrast, AST levels were significantly and similarly increased in both strains under CDA HFD (Fig 2E).

### Steatosis and liver inflammation in WT and *Pcolce*^-/-^ male mice under CDA HFD

**Fig 3:**
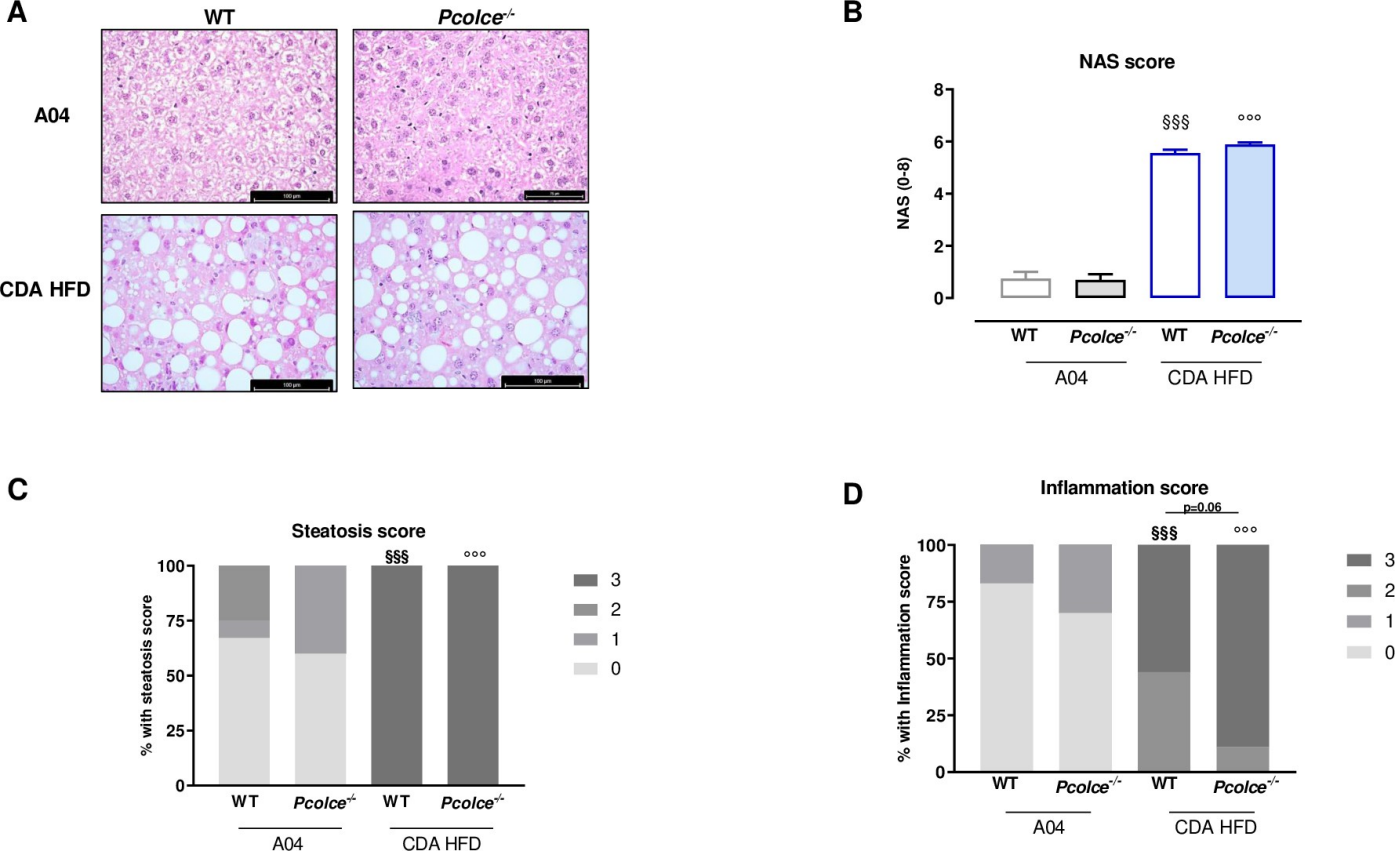
Liver steatosis and inflammation in WT and *Pcolce*^-/-^ male mice under A04 or CDA-HFD after 8 weeks. A: Typical examples of liver histology with Hematoxylin and Eosin staining for inflammation and steatosis analysis (bars represent 100 μm except *Pcolce*^*-/-*^ A04: 75 μm); B: evaluation of NAS score; C: steatosis score and D: inflammation score. Results are expressed as percentage of frequencies (C & D). NAS, steatosis and inflammation scores were determined as described in Material and Methods (n = 10–18). ^§§§^p<0.001 *vs*. WT A04; °°°p<0.001 *vs*. *Pcolce*^-/-^ A04. One-way ANOVA followed by Tukey’s post test for B. Fisher exact test for C & D.

Liver steatosis and inflammation were assessed by histology and NAS score was evaluated. As shown in Fig 3, CDA HFD induced a significant increase in both liver steatosis (Fig 3A and 3C), (with 100% of mice presenting more than 66% of affected hepatocytes) and inflammation (Fig 3D) in WT male mice resulting in a significant increase of NAS score (Fig 3B). While histological results were similar in *Pcolce*^-/-^ mice under CDA HFD (Fig 3A), a slight increase in inflammation score was nevertheless observed compared to WT male mice mainly driven by a slight increase of inflammatory foci observed in *Pcolce*^-/-^ mice (Fig 3D).

### Liver fibrosis in WT and *Pcolce*^-/-^ male mice under CDA HFD

**Fig 4:**
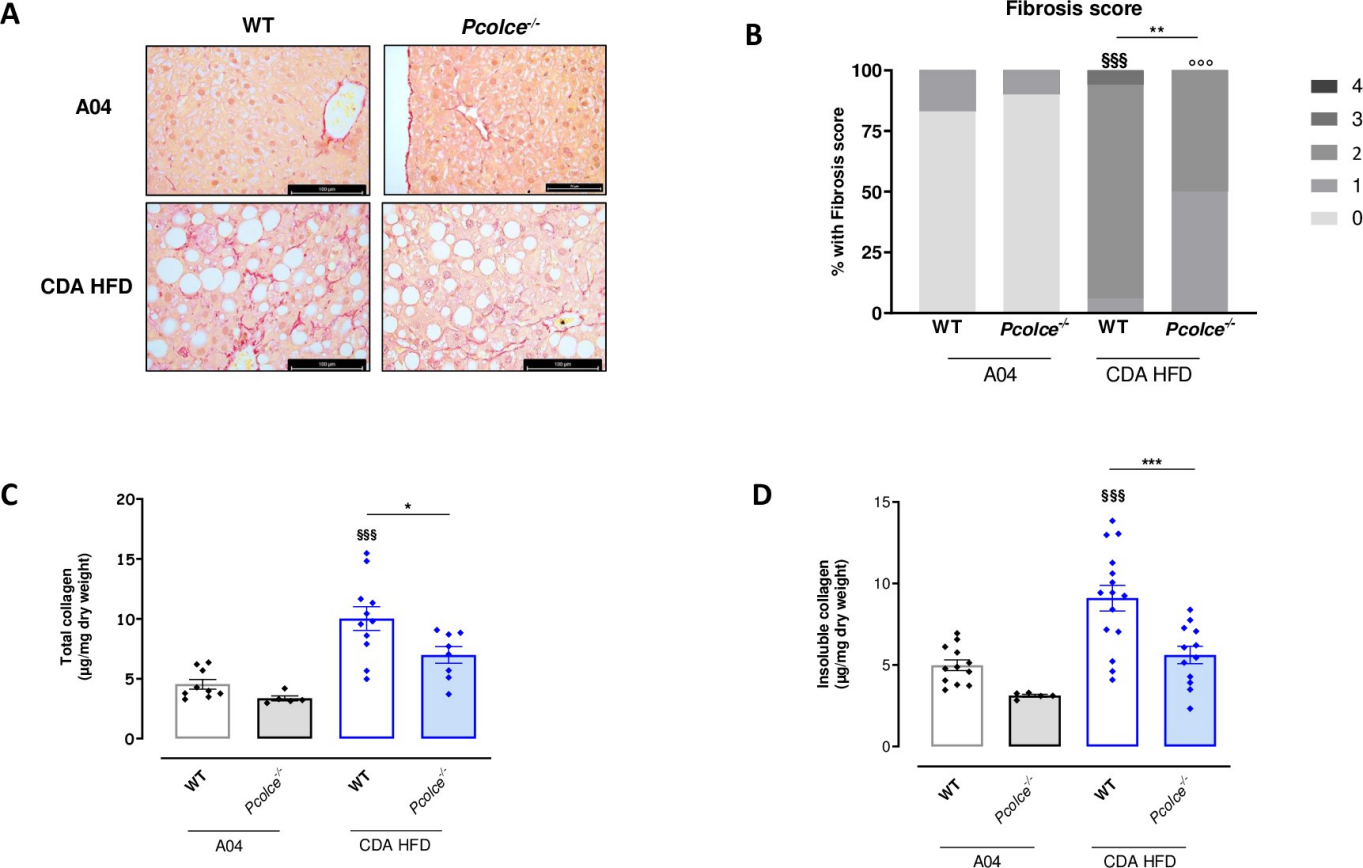
Liver fibrosis in WT and *Pcolce*^-/-^ male mice under A04 or CDA-HFD after 8 weeks. A: Typical examples of liver histology with Picrosirius red staining for fibrosis (bars represent 100 μm except *Pcolce*^*-/-*^ A04: 75 μm). B: Fibrosis score (n = 10–18). Fibrosis score was determined as described in Material and Methods and results are expressed as percentage of frequencies. ^§§§^p<0.001 *vs*. WT A04; °°°p<0.001 *vs*. *Pcolce*^-/-^ A04; **p<0.01 *vs*. WT CDA HFD. Fisher exact test. C: Total collagen content in liver (n = 5–11) and D: Insoluble collagen content in liver (n = 5–12). Data are expressed as mean ± SEM. ^§§§^p<0.001 *vs*. WT A04; *p<0.05, ***p<0.001 *vs*. WT CDA HFD. One-way ANOVA with Tukey’s post-test.

Fibrosis was first evaluated by histology with Picrosirius red staining of liver sections and scored. This scoring analysis was based on fibrosis localization (perisinusoidal, periportal fibrosis) or “structural” fibrosis (bridging fibrosis, septa, cirrhosis) as described in Material and Methods section. As expected, CDA HFD triggered liver fibrosis in WT male mice with the presence of both perisinusoidal and periportal fibrosis (Fig 4A and 4B). A significant decrease in liver fibrosis scoring was observed in *Pcolce*^-/-^ male mice compared to male WT under CDA HFD with a lower frequency of both perisinusoidal and periportal fibrosis (Fig 4B).

Total and insoluble liver collagen were then quantified. Total collagen was significantly increased in male WT under CDA HFD (Fig 4C). Under CDA HFD, PCPE-1 deficiency protected the mice, since significantly lower total collagen content was observed in *Pcolce*^-/-^ male mice than in WT male mice (Fig 4C). To evaluate the effect of *Pcolce* knock-out on collagen maturation in the CDA HFD model, liver insoluble collagen was also quantified. Liver insoluble collagen was increased in WT mice under CDA HFD, and PCPE-1 deficiency induced a significant decrease of cross-linked collagens compared to WT (Fig 4D). Collagen results are consistent with histological data (Fig 4A and 4B). Taken together, these results indicate that *Pcolce* gene deficiency limits CDA HFD-induced liver fibrosis.

### Liver mRNA expression of lipogenesis, inflammation and fibrosis genes in WT and *Pcolce*^-/-^ male mice under CDA HFD

**Fig 5:**
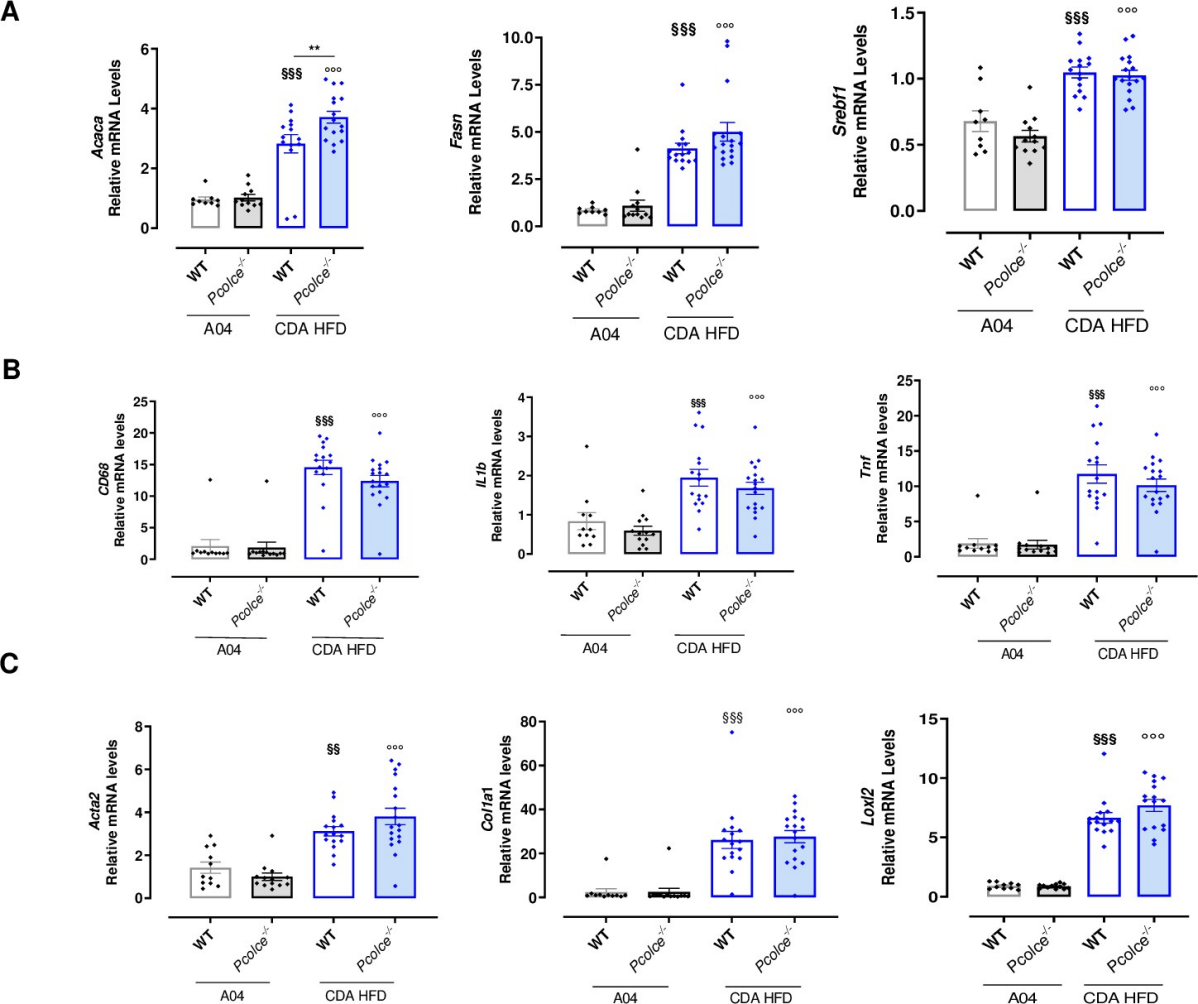
Liver mRNA expression of lipogenesis, inflammation and fibrosis genes in male mice under A04 or CDA HFD after 8 weeks. Gene expression by RT-qPCR analysis in WT and *Pcolce*^-/-^ male mice (n = 9–16). A: Fatty acid/ cholesterol synthesis (Acetyl-CoA carboxylase 1 (*Acaca*), Fatty Acid Synthase (*Fsn*), Sterol regulatory element-binding protein 1 (*Srebf1*)). B: Inflammation (*CD68*, Interleukin 1β (*Il1b)*, Tumor Necrosis Factor α (*Tnf*)). C: Fibrosis (Actin α2 (*Acta2*), procollagen type I α1 (*Col1a1*), Lysyl Oxidase-like-2 (*Loxl2*)). Data are expressed as mean ± SEM. ^§§§^p<0.001 *vs*. WT A04; °°°p<0.001 *vs*. *Pcolce*^-/-^ A04; **p<0.01 *vs*. WT CDA HFD. One-way ANOVA with Tukey’s post-test.

In order to better understand from a mechanistic standpoint, the impact of *Pcolce* gene deficiency on NASH progression and fibrosis, gene expression studies focusing on key relevant pathways were performed. As expected, CDA HFD induced an overexpression of genes involved in *de novo* lipogenesis (Acetyl-CoA carboxylase 1, Fatty Acid Synthase, Sterol Regulatory Element-Binding Protein 1) in both WT and *Pcolce*^-/-^ mice, with a slightly higher expression of Acetyl-CoA carboxylase 1 in *Pcolce*^-/-^ mice (Fig 5A). The increase of inflammation markers such as CD68 (monocyte/macrophage marker), Tumor Necrosis Factor-α (TNF-α) and Interleukin 1β (IL1-β) in CDA HFD model was similar in both strains (Fig 5B). Expression of fibrosis genes such as procollagen type I, Smooth Muscle Actin and Lysyl Oxidase Like-2 (LOXL2) was also similarly increased in WT and *Pcolce*^-/-^ mice under CDA HFD (Fig 5C).

These results indicate that *Pcolce* gene deficiency did not alter diet-induced expression of key genes involved in both NASH disease progression and fibrosis.

### Liver parameters in WT and *Pcolce*^-/-^ female mice under CDA HFD

CDA HFD model (8 weeks of diet) was used in WT and *Pcolce*^-/-^ female mice. As shown in S2A Fig, female mice (WT and *Pcolce*^-/-^) gradually gained body weight under CDA HFD. Liver weight and TG as well as ALT and AST levels were increased with the NASH diet, similarly in WT and *Pcolce*^-/-^ female mice (S2B–S2D Fig).

S3 Fig shows that CDA HFD induced a significant increase in both liver steatosis (S3A and S3C Fig) and inflammation (S3D Fig) in WT and *Pcolce*^-/-^ female mice resulting in a significant increase of the NAS score (S3B Fig).

Finally, CDA HFD induced liver fibrosis in WT and *Pcolce*^-/-^ female mice with the presence of both perisinusoidal and periportal fibrosis but no significant difference was observed between the 2 strains under NASH diet (S4A and S4B Fig). Interestingly, a slight decrease of liver fibrosis score was observed in *Pcolce*^-/-^ female mice compared to WT mice under control diet (S4B Fig). Total and insoluble collagen content were slightly but not significantly increased in WT mice under CDA HFD (S4C and S4D Fig). Under CDA HFD, *Pcolce*^-/-^ female mice showed a significant lower total and insoluble collagen content compared to WT mice (S4C and S4D Fig).

### Expression of PCPE-1 mRNA and protein in human liver specimens

**Fig 6:**
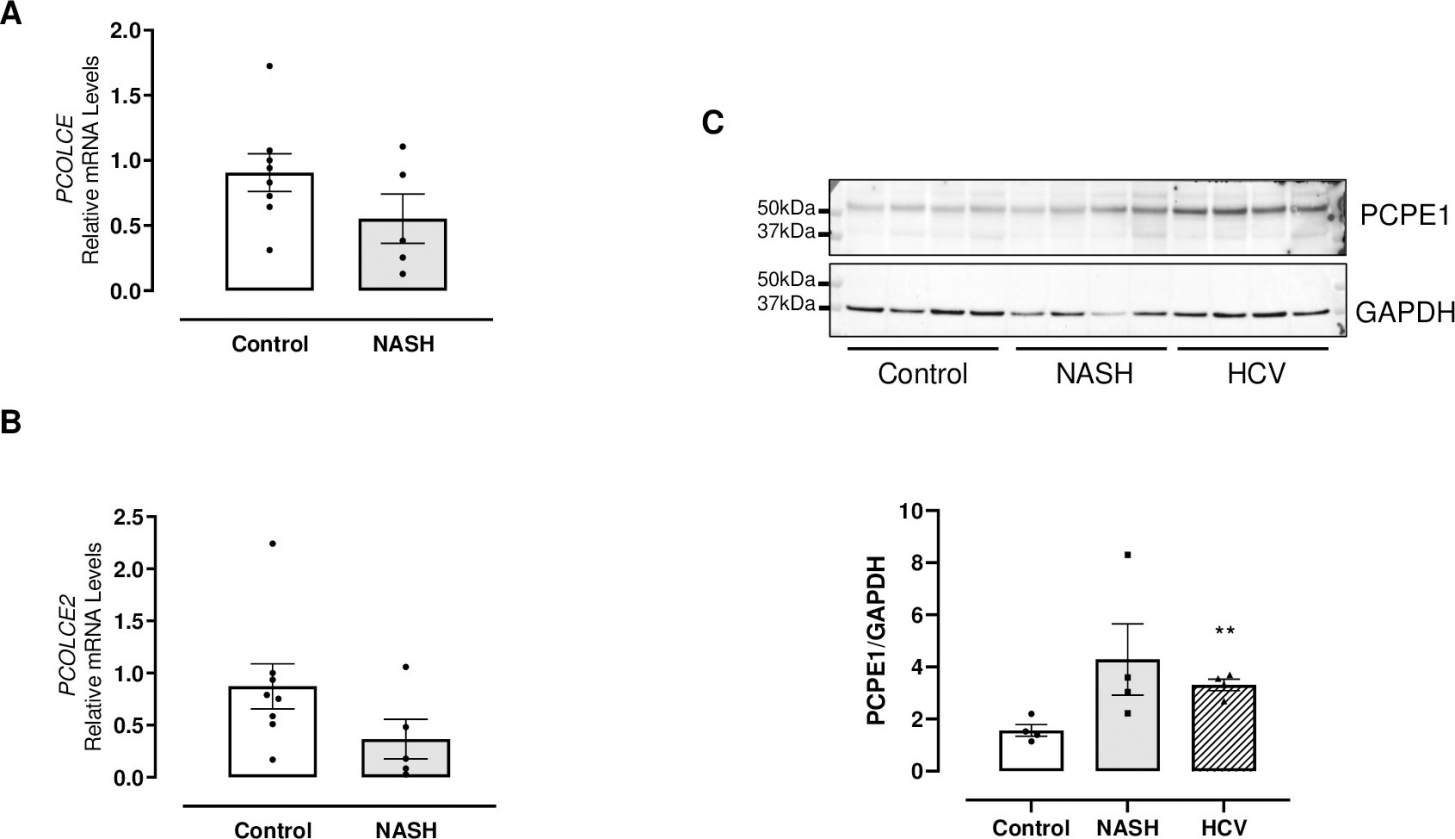
Expression of *PCOLCE* and *PCOLCE2* mRNA and PCPE-1 protein in human liver samples. *PCOLCE* (A) and *PCOLCE2* (B) mRNA expression in liver of control (n = 8) and NASH (F3-F4) patients (n = 5). PCPE-1 protein (C) in liver of control, NASH (F3-F4) and HCV cirrhotic patients (n = 4/group). One representative blot is shown, and the quantification was done from 3 independent experiments. Values reported from Western blots were obtained by band density analysis using Image Lab software (Bio-Rad) and expressed as the ratio of PCPE-1 / GAPDH. Data are expressed as mean ± SEM. **p<0.01 *vs*. control. Unpaired *t* test.

In order to assess the potential relevance of our preclinical findings, we next evaluated by RT-qPCR the hepatic expression of both *PCOLCE* and *PCOLCE2* in patients with NASH and fibrosis (F3-F4) (Table 1). Both genes were slightly less expressed in patients with NASH and fibrosis (Fig 6A and 6B, n = 8 and 5 for control and NASH respectively). This decrease was more pronounced for *PCOLCE2* even though it did not reach statistical significance. We next compared our results to previous published transcriptomic studies. Interestingly, results from 6 independent studies are in line with our findings (S1 Table). Finally, cirrhotic human liver samples were collected from NASH or HCV patients (Table 2) to evaluate PCPE-1 protein expression. All samples were scored by a pathologist. Samples without histological abnormalities were considered as controls (without steatosis, nor activity, nor fibrosis). Western blot analysis (Fig 6C, n = 4/group) showed that PCPE-1 protein expression was increased in livers of HCV patients (p<0.01) and slightly increased in liver of NASH patients but did not reach the statistical significance (p = 0.09).

## Discussion

This study describes the effect of PCPE-1 deficiency on liver fibrosis and NASH parameters in a murine model of NASH (CDA HFD). Liver PCPE-1 expression was also assessed in human livers of control and NASH or HCV related cirrhotic patients.

PCPE-1 is a key player of fibrosis as it is involved in fibrillar collagen maturation, critical step before collagen cross-linking, more resistant to degradation. Ogata *et al*. [20] have described that PCPE-1 is involved in the processing of type I collagen during liver fibrinogenesis under hepatic stress conditions such as murine CCl_4_ model. We thus investigated liver mRNA expression of *Pcolce* in different murine dietary models of NASH (STAM^TM^, Western Diet, GAN DIO and CDA HFD). Liver *Pcolce* mRNA expression was increased in all these models, confirming the induction of liver fibrosis markers in dietary models, but interestingly, the highest increase was observed in GAN DIO and CDA HFD models, two models associated with significant fibrosis [33]. Interestingly, PCPE-1 was recently identified as part of a gene cluster involved in ECM remodeling and directly linked to fibrosis by single cell transcriptomic analysis of HSCs from mice fed with Amylin diet [35] in line with our results. To investigate the role of PCPE-1 in liver fibrosis, the CDA HFD model (8 weeks) was selected to be used with *Pcolce*^-/-^ mice. Previous studies have shown that *Pcolce* knock-out mice (with different constructions) do not display any phenotypic abnormalities under basal conditions [25, 34], including blood chemical chemistry such as electrolytes, hepatic enzymes, total bilirubin, creatinine or urea [25]. As expected with choline deficient and methionine defined diet, male mice fed with CDA HFD had no body weight gain, as well as an increased liver weight and strong elevation of plasma ALT and AST [28]. PCPE-1 deficiency in male mice had no impact on body weight gain, ALT (slight decrease considered as non-relevant (even statistically significant)) and AST levels suggesting that this deficiency had no impact on diet-induced liver dysfunction. Liver steatosis, as well as mRNA expression of lipogenesis markers were highly induced in the model, with no significant effect of PCPE-1 deficiency. This result was expected, as modulation of fibrosis has no impact on cellular lipid accumulation driven by the diet. As expected, CDA HFD induced an increase of liver inflammation (histology and mRNA expression of typical markers such as CD68 (monocyte/macrophage) or cytokines (TNF-α, IL1-β)). PCPE-1 deficiency did not prevent this liver inflammation and even a slight increase of inflammatory foci was observed in liver of *Pcolce*^-/-^ mice under CDA HFD which could be attributed to an infiltration of neutrophils as observed by Massoudi *et al*. [36] in *Pcolce*^*-/-*^ mouse corneas after epithelial abrasion or alkali burn. Inflammatory response stimulates the transformation of quiescent HSCs into activated HSCs, which are the main source of collagens. Indeed, CDA HFD triggered liver fibrosis in WT mice with the presence of both perisinusoidal and periportal fibrosis, as well as bridging and septa fibrosis to a lesser extent. As expected, an increased mRNA expression of fibrosis markers such as Smooth Muscle Actin, procollagen type I and LOXL-2 was observed in WT mice under CDA HFD, leading to an increased level of total and insoluble collagen content. No liver weight increase was observed in *Pcolce*^-/-^ mice under NASH diet compared to mice under standard diet. This effect might be attributed, at least in part, to a beneficial effect on liver fibrosis, as no difference was observed in hepatic TG content. Indeed, PCPE-1 deficient mice under CDA HFD displayed a significant decrease of liver fibrosis with a lower frequency of both perisinusoidal and periportal fibrosis. This result was in accordance with a decreased liver collagen content associated with a decreased insoluble collagen observed in *Pcolce*^-/-^ mice under CDA HFD. This result suggests that, in this model, the maturation of procollagen into collagen fibers is dependent on PCPE-1 which enhances BMP-1 activity and accelerates collagen maturation and deposition [10]. PCPE-1 deficiency had no impact on CDA HFD-induced mRNA expression of Smooth Muscle Actin, procollagen type I and LOXL-2, this result suggests that PCPE-1 is a distal player and does not seem to be involved in the regulation of expression of these markers, at least in this model (with 8 weeks of a NASH diet). Taken together, these results suggest that PCPE-1 deficiency decreased liver fibrosis but had no impact on NASH progression in the CDA HFD model.

Although most of the NASH preclinical models including the CDA HFD murine model have been developed and characterized using male mice [28, 37] due to sexual dimorphism associated to NAFLD [38], we also carried out similar investigations in female mice. Female mice (WT and *Pcolce*^-/-^) gradually gained body weight under CDA HFD in contrast to males, as previously described [39]. In general, we found similar results in female mice with no impact on NASH progression and liver dysfunction upon *Pcolce* gene deletion. In contrast to male mice, *Pcolce* gene deficiency did not have a significant impact on CDA HFD-induced liver fibrosis in female mice as assessed by Picrosirius Red staining, even if no bridging fibrosis was observed in *Pcolce*^-/-^ female mice compared to WT female mice. However, we confirmed that *Pcolce* gene deficiency resulted in a significantly reduced total and insoluble collagen content upon CDA HFD similar to what was observed in male mice. Taken together, the data generated with female mice are consistent with the results obtained in males with an impact on liver fibrosis only.

A slight reduction in *PCOLCE2* expression was observed in human liver biopsies from NASH patients compared to control. Even though PCPE-2 has been shown to play a major role in HDL-cholesterol metabolism and more broadly in reverse cholesterol transport [14, 40], it could be also involved in liver fibrosis by a direct effect on procollagen maturation or an indirect effect *via* cholesterol metabolism. Interestingly, PCPE-2 is mainly expressed in the heart where its role in fibrosis has been established [31]. Further studies using PCPE-2 deficient mice are required to address this point.

Hassoun *et al*. [23] have shown that circulating PCPE-1 is increased in patients with liver fibrosis suggesting that PCPE-1 could be a key player of liver fibrosis in patients. We thus analyzed the expression of liver PCPE-1 in patients with NASH or HCV related cirrhosis. Expression of *PCOLCE* mRNA was not modified in patients compared to control (confirmed by published transcriptomic studies). PCPE-1 protein expression was slightly increased in NASH patients, but not significantly (p = 0.09) likely due to the small number of samples (n = 4/group). Why *PCOLCE* gene expression is not up regulated in patient derived samples in contrast to all tested preclinical models, is unclear. Further studies are needed to confirm these preliminary results. Moreover, it would be of interest to assess more precisely circulating PCPE-1 levels in NASH patients with various degrees of fibrosis (as reported by Gokce and colleagues [24] in patients suffering from chronic hepatitis B) to determine whether PCPE-1 could be a diagnostic biomarker or be used to monitor efficacy of anti-fibrotic drugs. The impact of aging should also be carefully analyzed since it is well-established that NASH disease progression and development of fibrosis are also function of age [41]. It has been hypothesized that fibrosis may be less prone to reverse due to impaired fibrolysis associated with increased cross-linking of collagen fibrils [8, 42]. The role of PCPE-1 in this process should be investigated in the future.

Circumstantial evidence links the degree of fibrosis to mortality in NASH patients [43]. Therefore, there is increasing interest in pharmacologic agents that can either reverse and/or slow down the progression of fibrosis. Since the pathogenesis of NASH is complex and involves multiple pathways, a combination of pharmacological agents may be required to tackle the problem rather than a single agent. Targeting the maturation of collagen *via* PCPE-1 antagonism, could be a relevant strategy to act on this mechanism. Further studies are needed to explore the combination of PCPE-1 deficiency with a drug treatment targeting inflammation/ steatosis to evaluate the impact on fibrosis and NASH progression.

## Supporting information

S1 Table *Pcolce* and *Pcolce2* expression in published transcriptomic analyses performed on NASH clinical samples. (DOCX)

S1 Fig Liver mRNA expression of *Pcolce*, *Pcolce2* and *Bmp1* in WT and *Pcolce* ^-/-^ male mice (18 week old, WT n = 9; *Pcolce*^-/-^ n = 12). Data are expressed as mean ± SEM. *p<0.05, ***p<0.001 *vs*. WT. Unpaired *t* test.
(TIF)

S2 Fig Body and liver weight, liver TG, ALT and AST levels in WT and *Pcolce*^-/-^ female mice under A04 or CDA HFD after 8 weeks. (A) Body weight gain during A04 or CDA HFD diet (n = 5–15). ^§§^p<0.05, °°°p<0.001 *vs*. WT A04. 2-way ANOVA followed by Tukey’s post-hoc analysis. (B) Liver weight (n = 5–15); (C) Liver TG content (n = 5–15); (D) Plasma Alanine Aminotransferase (ALT) level (n = 2 (*Pcolce*^-/-^ A04)-11); (E) Plasma Aspartate Aminotransferase (AST) level (n = 2 (*Pcolce*^-/-^ A04)-11). ^§§§^p<0.001 *vs*. WT A04; °°°p<0.001 *vs*. *Pcolce*^-/-^ A04. Panels B-E: One-way ANOVA with Tukey’s post hoc analysis. ^(1)^ For D and E, no statistical analyses and comparisons were done between *Pcolce*^*-/-*^ mice under A04 and CDA HFD as the number of WT mice under A04 diet was too low (n = 2). Data are expressed as mean ± SEM.
(DOCX)

S3 Fig Liver steatosis and inflammation in WT and *Pcolce*^-/-^ female mice under A04 or CDA-HFD after 8 weeks. (A) Typical examples of liver histology with Hematoxylin and Eosin staining for inflammation and steatosis analysis (bars represent 75 μm); (B) evaluation of NAS score; (C) steatosis score and (D) inflammation scores. Results are expressed as percentage of frequencies (C & D). NAS, steatosis and inflammation scores were determined as described in Material and Methods (n = 5–14). ^§§§^p<0.001 *vs*. WT A04; °°°p<0.001 *vs*. *Pcolce*^-/-^ A04. One-way ANOVA followed by Tukey’s post test for B. Fisher exact test for C & D.
(DOCX)

S4 Fig Liver fibrosis in WT and *Pcolce*^-/-^ female mice under A04 or CDA-HFD after 8 weeks. (A) Typical examples of liver histology with Picrosirius red staining for fibrosis (bars represent 75 μm). (B) Fibrosis score (n = 5–14). Fibrosis score was determined as described in Material and Methods and results are expressed as percentage of frequencies. ^§§^p<0.01 *vs*. WT A04; °°°p<0.001 *vs*. *Pcolce*^-/-^ A04. Fisher exact test. (C) Total collagen content in liver (n = 3–9) and (D) Insoluble collagen content in liver (n = 5–14). Data are expressed as mean ± SEM. *p<0.05 *vs*. WT CDA HFD. One-way ANOVA with Tukey’s post-test.
(DOCX)

S1 Raw images Western blotting for analysis of PCPE-1 protein expression in human liver samples. PCPE-1 and GAPDH protein in liver of control, NASH (F3-F4) and HCV cirrhotic patients (n = 4/group). Three independent experiments were done and are presented with Ponceau staining and with anti-PCPE1 and anti-GAPDH blotting.
(PDF)

S1 Raw dataset Evaluation of liver *Pcolce* mRNA gene expression in murine models of NASH (Fig 1). (PDF)

S2 Raw dataset Body (A) and liver weight (B), liver TG (C), ALT (D) and AST (E) levels in WT and *Pcolce*^-/-^ male mice under A04 or CDA HFD after 8 weeks (Fig 2).
(PDF)

S3 Raw dataset NAS score (A), liver steatosis (B) and inflammation (C) in WT and *Pcolce*^-/-^ male mice under A04 or CDA-HFD after 8 weeks (Fig 3).
(PDF)

S4 Raw dataset Liver fibrosis score (A), total collagen (B) and insoluble collagen (C) in WT and *Pcolce*^-/-^ male mice under A04 or CDA-HFD after 8 weeks (Fig 4).
(PDF)

S5 Raw dataset Liver mRNA expression of lipogenesis (A), inflammation (B) and fibrosis (C) genes in male mice under A04 or CDA HFD after 8 weeks (Fig 5).
(PDF)

S6 Raw dataset Expression of *PCOLCE* (A) and *PCOLCE2* (B) mRNA and PCPE-1 protein (C) in human liver samples (Fig 6).
(PDF)

S7 Raw dataset Liver mRNA expression of *Pcolce*, *Pcolce2* and *Bmp1* in WT and *Pcolce* ^-/-^ male mice (S1 Fig). (PDF)

S8 Raw dataset Body (A) and liver (B) weight, liver TG (C), ALT (D) and AST (E) levels in WT and *Pcolce*^-/-^ female mice under A04 or CDA HFD after 8 weeks (S2 Fig).
(PDF)

S9 Raw dataset NAS score (A), liver steatosis (B) and inflammation (C) in WT and *Pcolce*^-/-^ female mice under A04 or CDA-HFD after 8 weeks (S3 Fig).
(PDF)

S10 Raw dataset Liver fibrosis score (A), total collagen (B) and insoluble collagen (C) in WT and *Pcolce*^-/-^ female mice under A04 or CDA-HFD after 8 weeks (S4 Fig).
(PDF)
