# Supplementary material for: Procollagen C-Proteinase Enhancer-1 (PCPE-1) deficiency in mice reduces liver fibrosis but not NASH progression
Source: PLoS One. 2022 Feb 11;17(2):e0263828. doi: 10.1371/journal.pone.0263828 (PMC8836302; doi:10.1371/journal.pone.0263828)
Supplement: S1 Table — (DOCX) [file pone.0263828.s001.docx]

**S1 Table.** ***Pcolce* and *Pcolce2* expression in published transcriptomic analyses performed on NASH clinical samples**.

| **Study ID** | **Gene_ID** | **Log2 FC** | **Adjusted p value** |
| --- | --- | --- | --- |
|  |  |  |  |
| E-MEXP3291 | *Pcolce* | -0.008 | NS |
|  | *Pcolce2* | -1.39 | *** |
|  |  |  |  |
| GSE37031 | *Pcolce* | -0.242 | NS |
|  | *Pcolce2* | -0.194 | NS |
|  |  |  |  |
| GSE48452 | *Pcolce* | -0.241 | NS |
|  | *Pcolce2* | -0.255 | NS |
|  |  |  |  |
| GSE59045 | *Pcolce* | 0.543 | NS |
|  | *Pcolce2* | 0.151 | NS |
|  |  |  |  |
| GSE61260 | *Pcolce* | -0.427 | NS |
|  | *Pcolce2* | -0.186 | NS |
|  |  |  |  |
| GSE66676 | *Pcolce* | -0.199 | NS |
|  | *Pcolce2* | -0.055 | NS |
|  |  |  |  |
|  |  |  |  |

# NS: Not Significant. Log2 FC: Log2 Fold Change.

# ***p<0.001 Benjamin-Hochberg adjusted p values.
