# Supplementary figures and images for: Procollagen C-Proteinase Enhancer-1 (PCPE-1) deficiency in mice reduces liver fibrosis but not NASH progression
Source: PLoS One. 2022 Feb 11;17(2):e0263828. doi: 10.1371/journal.pone.0263828 (PMC8836302; doi:10.1371/journal.pone.0263828)

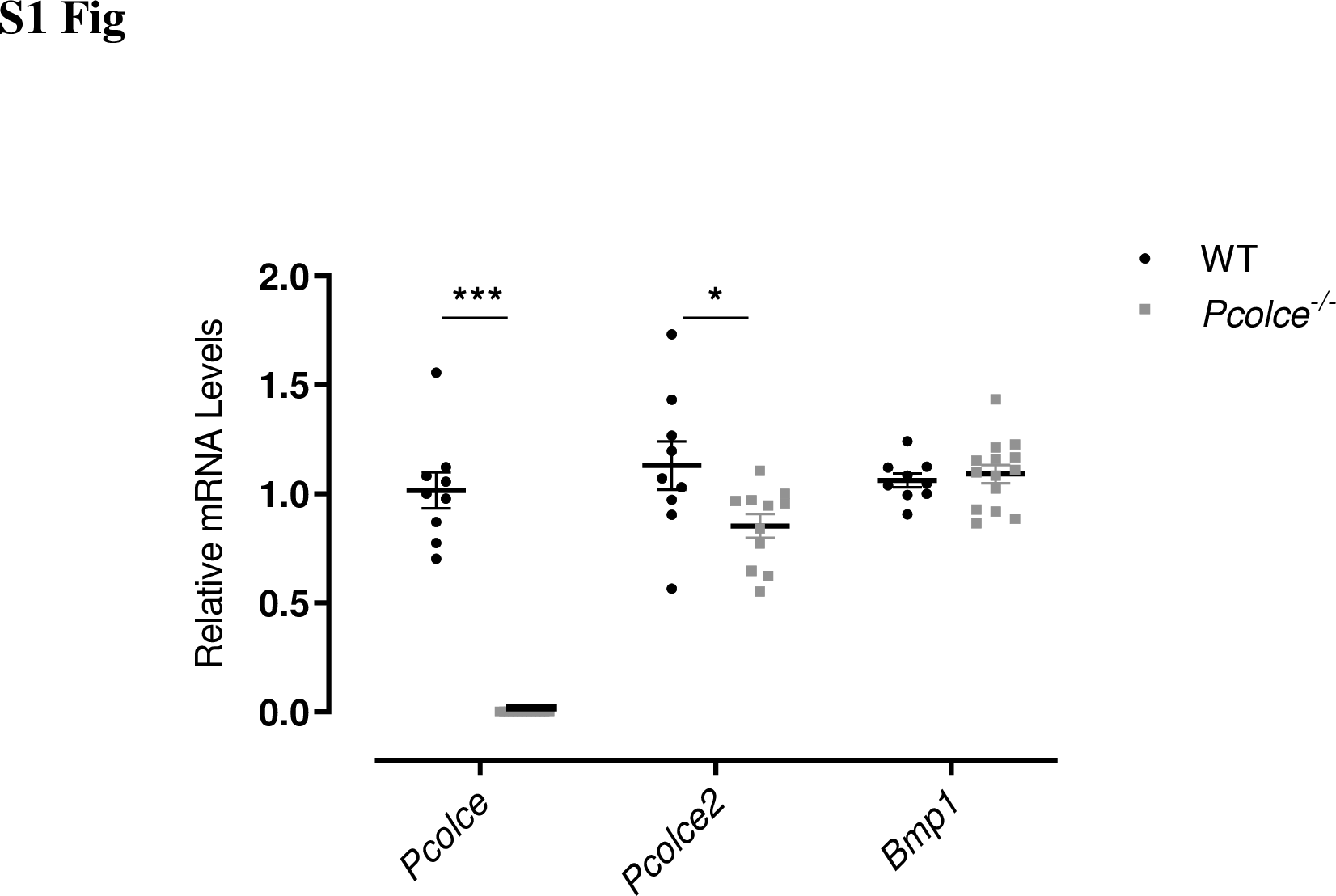

Supplement: S1 Fig — Data are expressed as mean ± SEM. *p<0.05, ***p<0.001 vs. WT. Unpaired t test. (TIF) [file pone.0263828.s002.tif]

**S3 Fig**

**A**

**WT**

**A04**

**CDA HFD**

***Pcolce-/-***


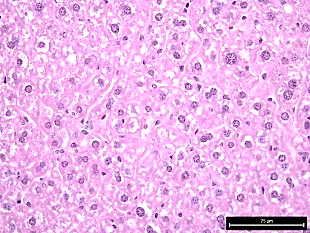

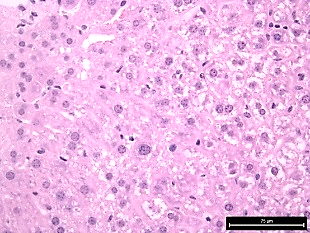

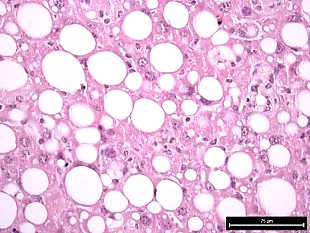

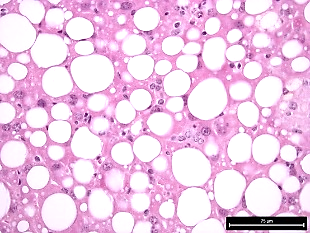


**B**

**C**

**D**

Supplement: S3 Fig — (A) Typical examples of liver histology with Hematoxylin and Eosin staining for inflammation and steatosis analysis (bars represent 75 μm); (B) evaluation of NAS score; (C) steatosis score and (D) inflammation scores. Results are expressed as percentage of frequencies (C & D). NAS, steatosis and inflammation scores were determined as described in Material and Methods (n = 5–14). §§§p<0.001 vs. WT A04; °°°p<0.001 vs. Pcolce-/- A04. One-way ANOVA followed by Tukey’s post test for B. Fisher exact test for C & D. (DOCX) [file pone.0263828.s004.docx]

**S4 Fig**

**A**

***Pcolce-/-***

**A04**

**CDA HFD**

**WT**


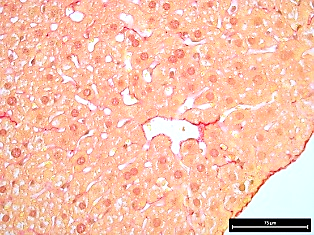

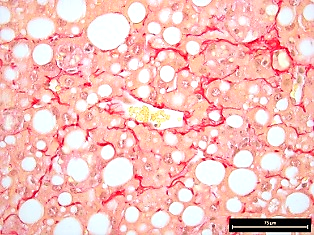

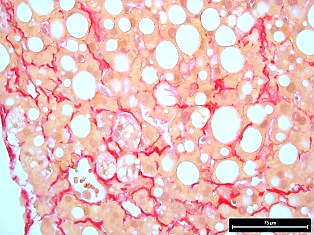

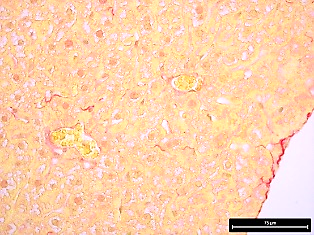


**B**

**C**

**D**

Supplement: S4 Fig — (A) Typical examples of liver histology with Picrosirius red staining for fibrosis (bars represent 75 μm). (B) Fibrosis score (n = 5–14). Fibrosis score was determined as described in Material and Methods and results are expressed as percentage of frequencies. §§p<0.01 vs. WT A04; °°°p<0.001 vs. Pcolce-/- A04. Fisher exact test. (C) Total collagen content in liver (n = 3–9) and (D) Insoluble collagen content in liver (n = 5–14). Data are expressed as mean ± SEM. *p<0.05 vs. WT CDA HFD. One-way ANOVA with Tukey’s post-test. (DOCX) [file pone.0263828.s005.docx]
