# Supplementary material for: Procollagen C-Proteinase Enhancer-1 (PCPE-1) deficiency in mice reduces liver fibrosis but not NASH progression
Source: PLoS One. 2022 Feb 11;17(2):e0263828. doi: 10.1371/journal.pone.0263828 (PMC8836302; doi:10.1371/journal.pone.0263828)
Supplement: S2 Fig — (A) Body weight gain during A04 or CDA HFD diet (n = 5–15). §§p<0.05, °°°p<0.001 vs. WT A04. 2-way ANOVA followed by Tukey’s post-hoc analysis. (B) Liver weight (n = 5–15); (C) Liver TG content (n = 5–15); (D) Plasma Alanine Aminotransferase (ALT) level (n = 2 (Pcolce-/- A04)-11); (E) Plasma Aspartate Aminotransferase (AST) level (n = 2 (Pcolce-/- A04)-11). §§§p<0.001 vs. WT A04; °°°p<0.001 vs. Pcolce-/- A04. Panels B-E: One-way ANOVA with Tukey’s post hoc analysis. (1) For D and E, no statistical analyses and comparisons were done between Pcolce-/- mice under A04 and CDA HFD as the number of WT mice under A04 diet was too low (n = 2). Data are expressed as mean ± SEM. (DOCX) [file pone.0263828.s003.docx]

**S2 Fig**

**A**

**B**

**C**

**D**

**E**
