# Supplementary material for: Procollagen C-Proteinase Enhancer-1 (PCPE-1) deficiency in mice reduces liver fibrosis but not NASH progression
Source: PLoS One. 2022 Feb 11;17(2):e0263828. doi: 10.1371/journal.pone.0263828 (PMC8836302; doi:10.1371/journal.pone.0263828)
Supplement: S1 Raw images — PCPE-1 and GAPDH protein in liver of control, NASH (F3-F4) and HCV cirrhotic patients (n = 4/group). Three independent experiments were done and are presented with Ponceau staining and with anti-PCPE1 and anti-GAPDH blotting. (PDF) [file pone.0263828.s006.pdf]

Fig 6: Gel #1 (image captured by ChemiDoc imaging system (Bio-Rad)).

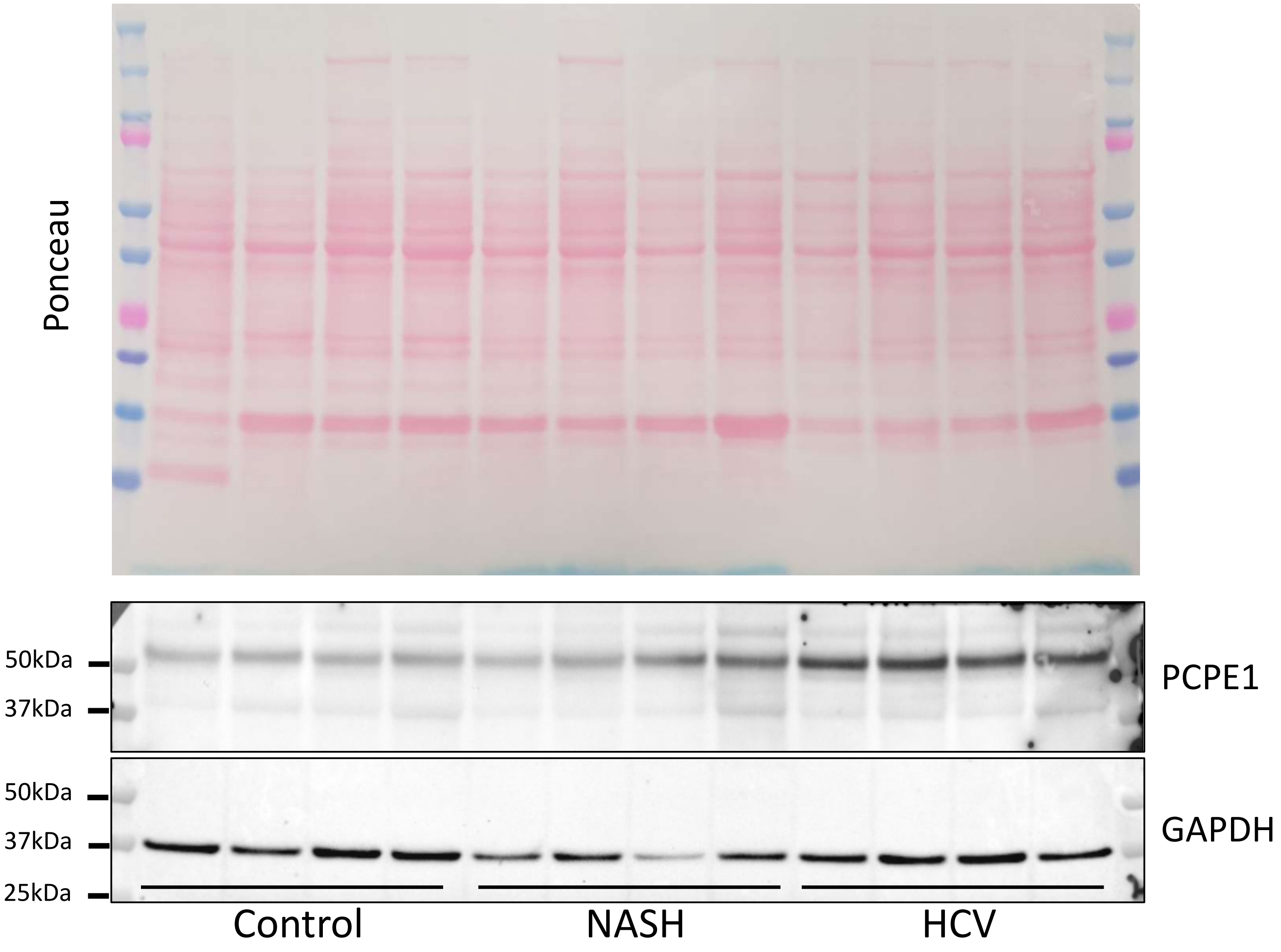

Fig 6: Gel #2 (image captured by ChemiDoc imaging system (Bio-Rad)).

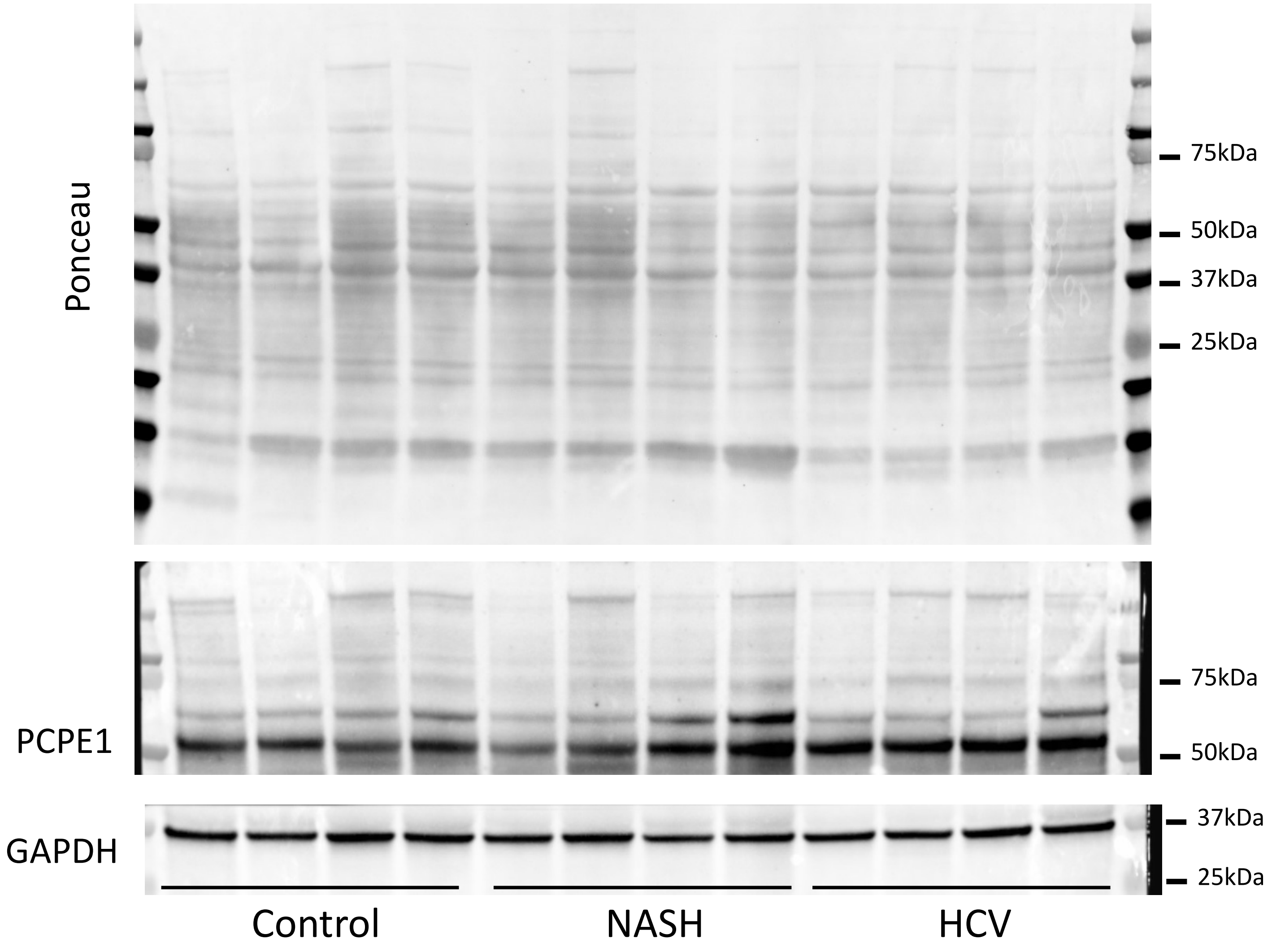

Fig 6: Gel #3 (image captured by ChemiDoc imaging system (Bio-Rad)).

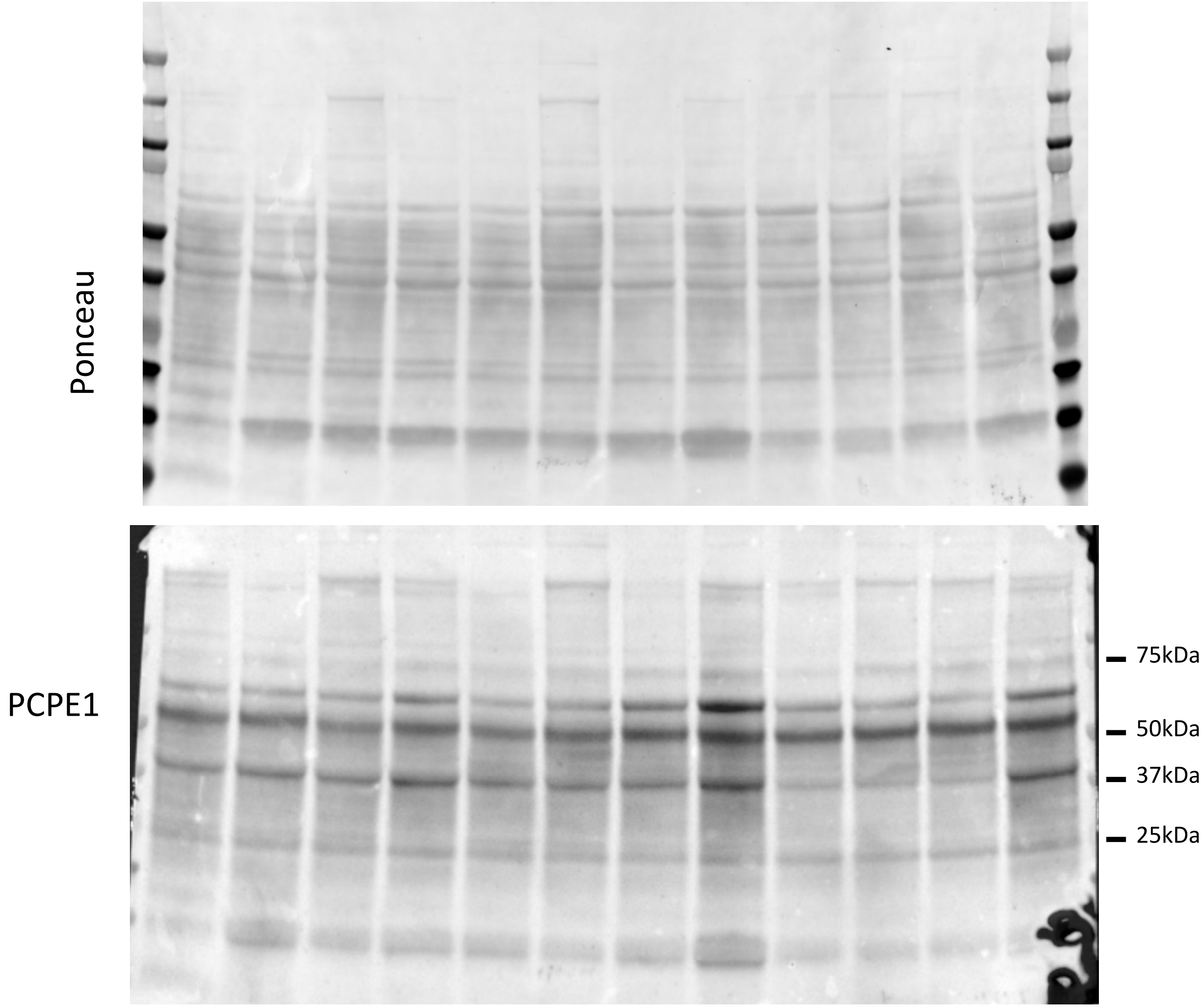

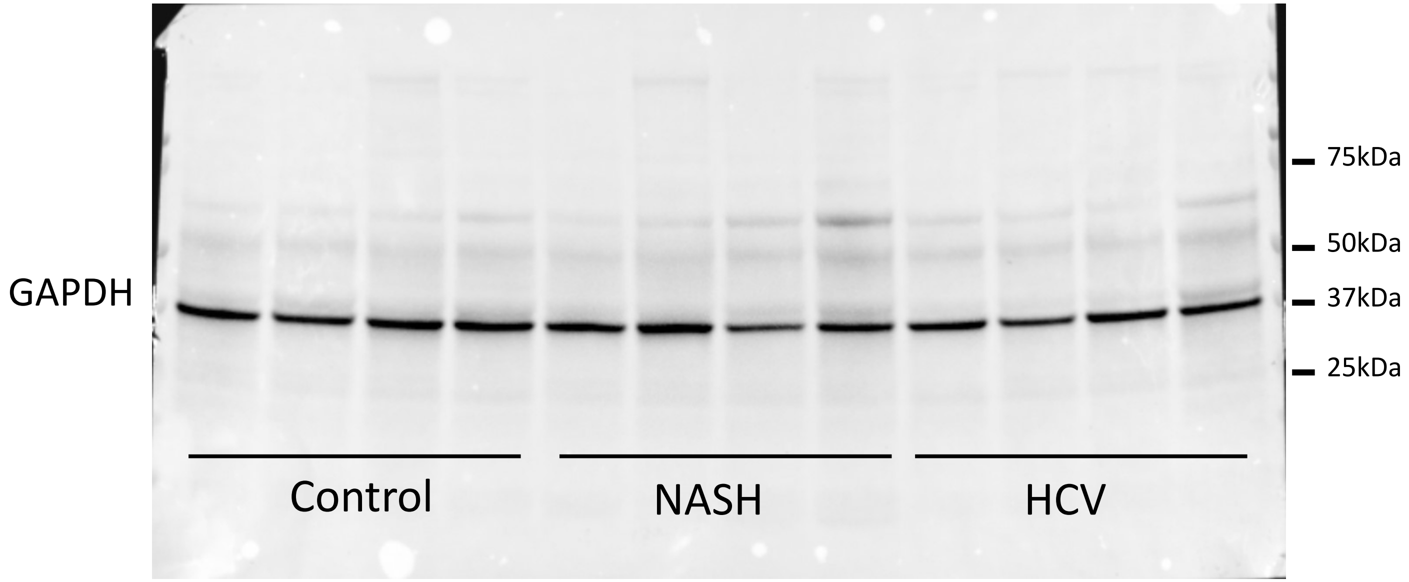
GAPDH
75kDa
50kDa
37kDa
25kDa
Control
NASH
HCV
