# Supplementary material for: Procollagen C-Proteinase Enhancer-1 (PCPE-1) deficiency in mice reduces liver fibrosis but not NASH progression
Source: PLoS One. 2022 Feb 11;17(2):e0263828. doi: 10.1371/journal.pone.0263828 (PMC8836302; doi:10.1371/journal.pone.0263828)
Supplement: S1 Raw dataset — (PDF) [file pone.0263828.s007.pdf]

A

Control	STAM
0,67	1,62
0,94	1,15
1,06	1,16
0,92	1,88
	1,69
	1,95

B

Control	Western Diet
0,81	1,53
1,12	1,12
1,04	1,04
1,04	1,62
0,9	1,71
0,92	1,15
0,97	0,8
1,22	1,46
0,89	1,46
1,09	

C

Control	GAN DIO
0,98	3,39
1,22	3,36
0,80	4,15

D

Control	CDA HFD
0,91	5,16
0,98	3,72
1,05	2,85
1,27	3,79
0,77	3,52
1,09	4,06
1,06	4,19
1,11	5,32
0,91	6,45
0,85	3,94
