# Supplementary material for: Procollagen C-Proteinase Enhancer-1 (PCPE-1) deficiency in mice reduces liver fibrosis but not NASH progression
Source: PLoS One. 2022 Feb 11;17(2):e0263828. doi: 10.1371/journal.pone.0263828 (PMC8836302; doi:10.1371/journal.pone.0263828)
Supplement: S2 Raw dataset — Body (A) and liver weight (B), liver TG (C), ALT (D) and AST (E) levels in WT and Pcolce-/- male mice under A04 or CDA HFD after 8 weeks (Fig 2). (PDF) [file pone.0263828.s008.pdf]

A

Time (w)	WT A04												
0	25,2	25,4	23,1	25	26,9	25,6	24,4	21,89	20,39	23,09	23,37	22,62	25,07
1	25,7	25,6	24,9	25,2	28,5	26,4	25	21,89	20,63	23,15	24,46	24,32	25,17
2	26,5	25,8	26,2	27,6	31,7	29	26,7	23,15	21,86	24,27	24,78	26,53	26,32
3	27,6	27	25,03	26,25	30,53	27,92	26,59	25,1	22,87	25,28	25,76	28,08	27,51
4	27,64	27,99	26,14	26,96	31,37	29,4	28,12	25,93	23,51	27,24	25,56	29,7	27,64
5	29,5	28,91	27,51	27,72	32,31	30,27	29,05	26,36	24,55	27,98	25,38	30,39	29,89
6	30,08	29,83	27,45	28,04	32,82	31,44	30,5	27,05	24,19	29,98	25,94	31,48	30,73
7	31,24	30,8	29,04	28,98	34,17	32,51	32,21	28,25	25,35	31,01	26,87	32,68	31,77
8	32,77	31,61	30,9	30,34	34,88	32,85	33,53	29,42	26,54	32,3	27,71	32,81	33,05

Time (w)	*Pcolce*$^{-/-}$ A04											
0	23,3	22,32	19,21	15,23	24,39	22,91	23,59	22,81	23,21	25,19	22,71	23,66
1	24,59	22,32	24,84	23,26	25,2	24,28	23,7	23,16	24,94	25,92	23,99	24,16
2	24,94	22,77	26,15	24,43	26,79	25,35	24,77	24,34	25,81	27,42	25,34	24,77
3	26,75	24,62	26,32	24,74	28,34	26,49	25,51	25,28	26,78	29,18	25,3	24,89
4	27,78	26,15	26,58	24,78	29,69	27,22	26,17	25,62	27,77	29,49	26,35	25,53
5	28,48	27,25	27,85	25,97	31,07	29,19	27,2	26,71	28,72	30,36	27,47	26,44
6	29,95	28,46	28,91	26,19	32,58	30,9	28,15	27,24	29,6	31,39	29,29	28,21
7	30,91	30,11	30	26,7	33,4	32,19	29,22	28,89	29,95	32,24	30,22	29,02
8	32,23	31	31,26	27,46	33,6	33,81	30,11	29,64	30,22	33,09	32,27	30,91

Time (w)	WT CDA HFD															
0	25,1	25,9	25,5	27,7	27	25,3	24,8	25,2	22,9	22,31	21,4	24,39	25,43	21,35	24,82	22,08
1	25,5	26,3	26,5	25,6	26,4	24,4	25,2	24,8	22,5	21,9	20,73	24,16	25,31	20,2	20,16	21,95
2	24,6	25,4	25,9	25,8	26,2	23,2	25,2	24,3	21,89	22,84	21,16	23,45	24,75	19,48	19,14	21,94
3	24,7	25,6	26,2	25,65	26,04	22,27	24,56	24,2	21,89	22,98	21,7	23,83	25,23	20,59	21,65	22,47
4	24,49	25,42	26,61	25,9	26,34	22,44	24,38	24,5	22,51	22,29	21,71	23,78	24,9	21,08	22,24	22,95
5	24,58	25,58	26,95	26,06	27,41	22,13	24,21	24,72	22,49	22,37	22,33	24,31	25,87	21,54	23,47	22,97
6	25,01	25,51	27,26	26,47	26,71	22,35	24,26	24,85	23,21	22,55	22,42	24,61	26,53	21,97	23,24	23,77
7	24,75	25,83	27,75	26,85	27,14	22,87	24,62	24,91	23,45	22,47	22,84	24,54	26,84	22,3	23,89	24,08
8	24,73	25,92	27,87	27,56	27,42	23,15	24,52	25,41	23,81	23,12	23,27	24,44	27,87	22,8	23,34	24,81

Time (w)	*Pcolce*$^{-/-}$ CDA HFD																	
0	21,4	20,7	20,95	21,81	19,14	24,39	20,35	21,78	21,05	21,79	24,53	21,47	24,47	23,89	23,8	24,3	23,15	22,48
1	21,5	20,2	19,03	20,41	19,15	22,5	21,67	20,97	21,87	21,26	22,96	23,24	23,68	23,5	22,5	22,78	23,31	23,36
2	21,78	21,01	19,01	19,38	19,05	21,76	21,08	20,72	22,84	21,3	23,88	23,53	24,38	23,65	22,06	22,66	22,38	22,88
3	21,96	21,09	19,22	19,05	19,31	21,06	20,64	20,89	23,51	21,01	22,47	22,01	22,61	21,91	21,57	22,72	21,85	23,41
4	22,64	21,77	19,56	19,31	20,28	20,58	21,02	20,76	23,23	20,66	21,71	21,42	23,35	22,57	22,13	23,15	22,03	23,14
5	23,26	21,97	19,25	19,21	21,16	20,7	21,05	21,01	22,06	20,98	21,96	22,08	23,32	22,94	22,2	22,92	21,9	23,26
6	22,94	21,62	20,04	19,59	20,84	20,96	21,11	21,2	21,6	21,31	21,98	22,12	23,47	23,47	22,93	23,03	22,73	23,68
7	23,53	21,58	19,82	19,54	21,92	21,24	21,57	21,52	21,56	21,79	22,59	22,39	23,77	23,73	23,61	23,47	22,85	24,67
8	24	21,57	19,34	20,34	21,67	21,37	21,6	21,47	21,92	22,09	23,19	23,07	23,73	24	23,74	24,29	23,33	24,78

B

WT A04												
1,44	1,02	1,35	1,18	1,55	1,55	1,49	0,97	1,17	1,38	1,03	1,34	1,27

Pcolce$^{-/-}$ A04											
1,39	1,32	1,27	1,02	1,24	1,33	1,23	1,19	1,31	1,38	1,38	1,29

WT CDA HFD															
1,54	1,78	2,07	2,13	2,05	1,43	1,65	1,88	1,57	1,63	1,58	1,58	2,23	1,45	1,11	1,55

Pcolce$^{-/-}$ CDA HFD																
1,62	1,34	0,83	1,46	0,89	1,24	1,29	1,33	1,25	1,32	1,32	1,29	1,50	1,53	1,54	1,45	1,64

C

WT A04												
31,4	26,5	44,4	20,5	20,8	18,3	29,5	12,3	15,6	29,2	20,9	27,9	38,3

Pcolce$^{-/-}$ A04										
24,9	22,5	23,7	12,7	28,7	30,5	14,8	24,4	20,5	18,3	20

WT CDA HFD															
150,7	157	183,5	153,6	163,2	160,2	195,5	203,3	170,4	152,4	213,5	131,6	189,7	197,3	192,9	174,3

Pcolce$^{-/-}$ CDA HFD															
176,3	150,4	167,2	216,8	174,8	136,8	182,8	215,1	196,6	163,3	174,5	198,8	139,2	160,7	263	149,2

D

WT A04											
29	38	41	82	95	64	49	44	38	22	30	33

Pcolce$^{-/-}$ A04							
30	26	26	29	36	32	25	34

WT CDA HFD													
411	431	490	497	377	330	551	485	417	431	376	468	554	311

Pcolce$^{-/-}$ CDA HFD														
372	395	427	328	346	317	361	395	429	376	415	289	352	455	501

E

WT A04										
58	76	207	100	117	152	74	86	97	66	95

Pcolce$^{-/-}$ A04								
88	57	109	89	106	94	87	83	81

WT CDA HFD													
341	336	427	388	653	840	416	417	358	442	349	362	411	559

Pcolce$^{-/-}$ CDA HFD														
268	252	1058	897	382	782	430	335	393	396	333	296	245	357	421
