# Supplementary material for: Procollagen C-Proteinase Enhancer-1 (PCPE-1) deficiency in mice reduces liver fibrosis but not NASH progression
Source: PLoS One. 2022 Feb 11;17(2):e0263828. doi: 10.1371/journal.pone.0263828 (PMC8836302; doi:10.1371/journal.pone.0263828)
Supplement: S3 Raw dataset — NAS score (A), liver steatosis (B) and inflammation (C) in WT and Pcolce-/- male mice under A04 or CDA-HFD after 8 weeks (Fig 3). (PDF) [file pone.0263828.s009.pdf]

A

**WT A04**	***Pcolce***$^{-/-}$ **A04**	**WT CDA HFD**	***Pcolce***$^{-/-}$ **CDA HFD**
0	0	6	6
1	1	6	6
0	1	5	6
2	1	6	6
2	0	6	5
0	0	6	6
0	1	5	6
1	1	5	6
0	0	6	6
0	2	5	6
1		5	6
2		6	6
		6	6
		5	6
		6	6
		5	6
			6
			5

B

	**0**	**1**	**2**	**3**
**WT A04**	67	8	25	0
Pcolce$^{-/-}$ **A04**	60	40	0	0
**WT CDA HFD**	0	0	0	100
Pcolce$^{-/-}$ **CDA HFD**	0	0	0	100

C

	**0**	**1**	**2**	**3**
**WT A04**	83	17	0	0
Pcolce$^{-/-}$ **A04**	70	30	0	0
**WT CDA HFD**	0	0	44	56
Pcolce$^{-/-}$ **CDA HFD**	0	0	11	89
