# Supplementary material for: Procollagen C-Proteinase Enhancer-1 (PCPE-1) deficiency in mice reduces liver fibrosis but not NASH progression
Source: PLoS One. 2022 Feb 11;17(2):e0263828. doi: 10.1371/journal.pone.0263828 (PMC8836302; doi:10.1371/journal.pone.0263828)
Supplement: S4 Raw dataset — Liver fibrosis score (A), total collagen (B) and insoluble collagen (C) in WT and Pcolce-/- male mice under A04 or CDA-HFD after 8 weeks (Fig 4). (PDF) [file pone.0263828.s010.pdf]

A

	0	1	2	3	4
**WT A04**	83	17	0	0	0
***Pcolce$^{-/-}$* A04**	90	10	0	0	0
**WT CDA HFD**	0	6	88	6	0
***Pcolce$^{-/-}$* CDA HFD**	0	50	50	0	0

B

WT A04	*Pcolce$^{-/-}$* A04	WT CDA HFD	*Pcolce$^{-/-}$* CDA HFD
6,36	3,14	11,67	7,71
3,77	3,22	8,61	5,11
3,49	4,19	9,8	3,71
4,29	2,98	5,67	5,69
3,79	3,3	9,56	8,71
3,99		10,44	9,07
5,69		14,82	7,04
6,21		11,34	8,84
3,29		15,48	
		7,9	
		4,98	

C

WT A04	*Pcolce$^{-/-}$* A04	WT CDA HFD	*Pcolce$^{-/-}$* CDA HFD
3,47	3,3	10,07	6,21
5,77	3,26	12,98	5,15
4,6	2,84	13,85	6,08
4,78	3,1	8,41	3,5
6,12	3,11	7,17	7,76
4,88		4,09	7,27
3,74		9,44	5,46
5,14		9,43	3,92
4,05		9,25	7,07
6,95		11,27	2,33
6,57		7,04	4,29
3.79		10,62	8,39
		13,05	
		5,23	
		4,62	
