# Supplementary material for: Procollagen C-Proteinase Enhancer-1 (PCPE-1) deficiency in mice reduces liver fibrosis but not NASH progression
Source: PLoS One. 2022 Feb 11;17(2):e0263828. doi: 10.1371/journal.pone.0263828 (PMC8836302; doi:10.1371/journal.pone.0263828)
Supplement: S5 Raw dataset — Liver mRNA expression of lipogenesis (A), inflammation (B) and fibrosis (C) genes in male mice under A04 or CDA HFD after 8 weeks (Fig 5). (PDF) [file pone.0263828.s011.pdf]

A

Acaca

WT A04	$Pcolce^{-/-}$ A04	WT CDA HFD	$Pcolce^{-/-}$ CDA HFD
0.895	0,768	0,378	3,246
1	0,94	0,308	2,558
0,84	0,585	3,461	4,33
0,808	0,891	3,301	4,343
1,579	1,243	3,594	3,344
0,968	0,805	2,779	4,983
0,931	1,138	2,809	4,855
0,761	1,769	3,041	4,204
0,844	0,84	3,387	2,904
	0,78	2,981	3,405
	1,48	2,614	4,842
		4,12	3,209
		3,908	3,816
		2,875	3,615
			2,786
			2,938

Fasn

WT A04	$Pcolce^{-/-}$ A04	WT CDA HFD	$Pcolce^{-/-}$ CDA HFD
0.93	0,47	4,64	4,00
1	0,63	3,49	3,27
0,87	0,48	3,44	9,81
0,78	4,07	3,98	9,57
1,26	0,64	3,49	4,76
0,76	1,10	4,36	7,70
0,70	0,54	3,79	4,93
0,61	0,91	3,50	3,37
0,76	1,82	4,34	3,69
	0,61	3,81	3,94
	0,65	3,65	3,58
	1,25	3,08	4,90
		5,03	4,27
		7,51	4,39
		3,92	4,54
			4,35
			4,18

Srebpf1

WT A04	$Pcolce^{-/-}$ A04	WT CDA HFD	$Pcolce^{-/-}$ CDA HFD
0,54	0,45	1,14	0,99
1	0,48	1,15	1,15
1,08	0,46	1,04	0,76
0,75	0,93	1,11	0,78
0,67	0,48	1,34	1,16
0,49	0,63	1,27	0,84
0,69	0,53	1,10	1,12
0,45	0,56	0,86	0,91
0,43	0,67	0,86	1,30
	0,36	1,13	0,96
	0,70	0,99	0,98
	0,52	0,90	1,08
		1,07	1,00
		0,77	1,32
		0,95	1,07
			0,99
			1,01

B

Cd68

WT A04	*Pcolce*$^{-/-}$ A04	WT CDA HFD	*Pcolce*$^{-/-}$ CDA HFD
1	13,50	0,94	13,60
1,36	19,11	0,86	14,16
12,59	13,86	0,71	11,57
0,90	1,33	0,62	10,67
1,10	18,54	12,37	10,24
0,97	14,40	0,80	9,75
1,08	14,59	0,89	0,84
0,94	17,74	1,19	14,35
0,96	16,46	1,10	13,70
0,92	15,67	0,94	15,40
1,21	16,16	1,25	11,68
	15,38	1,16	12,35
	19,51	1,04	8,63
	8,16		15,66
	16,46		19,99
	11,85		13,49
			11,89
			14,93

Il1b

WT A04	*Pcolce*$^{-/-}$ A04	WT CDA HFD	*Pcolce*$^{-/-}$ CDA HFD
1	1,48	0,37	1,18
2,75	1,54	0,38	1,91
1,34	1,49	0,13	1,28
0,22	0,63	0,13	1,07
0,62	1,90	1,62	1,78
0,45	1,39	0,52	0,91
0,98	1,10	0,23	0,45
0,63	1,30	0,59	1,17
0,48	2,03	0,5	1,16
0,48	2,32	0,84	1,88
0,24	1,28	0,98	2,34
	2,44	0,88	2,22
	3,29	0,55	1,68
	2,08		2,38
	3,61		3,23
	3,25		2,02
			1,94
			1,57

Tnf

WT A04	*Pcolce*$^{-/-}$ A04	WT CDA HFD	*Pcolce*$^{-/-}$ CDA HFD
1	8,32	1	7,56
1,67	11,13	0,73	12,11
8,66	9,23	0,54	8,57
0,75	1,89	0,61	7,45
1,37	14,05	9,16	11,17
0,88	8,84	0,84	6,34
1,61	6,94	0,78	0,71
1,52	8,65	1,48	8,15
1,28	13,64	1,36	9,19
1,14	13,38	1,22	12,61
0,89	7,65	1,92	13,65
	16,09	1,16	12,15
	21,37	1,66	8,56
	9,29		14,12
	18,83		17,34
	18,61		13,18
			11,40
			8,26

C

Acta2

WT A04	*Pcolce$^{-/-}$* A04	WT CDA HFD	*Pcolce$^{-/-}$* CDA HFD
1	2,67	1,39	2,76
2,42	2,99	0,65	2,63
2,90	3,30	0,70	5,10
0,93	1,56	0,60	6,41
1,90	3,35	2,91	2,87
2,48	2,91	0,41	5,78
1,6	2,73	0,61	0,57
0,69	2,96	0,88	2,50
0,44	3,39	0,94	3,17
0,72	3,60	0,96	5,10
0,56	2,40	1,26	3,03
	3,79	0,69	4,76
	4,71	1,00	2,02
	1,98		6,24
	4,92		4,19
	2,65		3,77
			3,39
			3,32

Col1a1

WT A04	*Pcolce$^{-/-}$* A04	WT CDA HFD	*Pcolce$^{-/-}$* CDA HFD
1	17,81	1,26	15,84
1,22	31,79	0,47	23,04
17,48	18,42	0,46	36,53
0,89	1,40	0,47	46,05
1,09	30,6	22,33	15,62
1,77	20,16	0,30	39,32
1,02	21,83	0,34	0,80
0,39	24,39	0,80	21,28
0,38	26,27	1,19	27,02
0,74	30,39	0,83	31,96
0,67	25,38	1,46	17,41
	28,95	0,60	35,59
	36,10	1,35	13,61
	11,57		29,82
	75,13		42,92
	17,96		35,49
			32,37
			32,48

Loxl2

WT A04	*Pcolce$^{-/-}$* A04	WT CDA HFD	*Pcolce$^{-/-}$* CDA HFD
1	1,08	6,10	6,01
1,30	0,65	7,9	5,71
0,81	0,62	5,48	10,17
1,29	0,77	6,67	10,48
1,14	0,63	5,56	4,76
1	0,73	6,37	9,75
0,57	0,99	6,58	5,84
0,67	0,94	6,22	8,10
0,72	0,92	6,30	5,65
0,64	1,21	6,37	8,47
	0,85	7,09	4,42
	1,06	7,17	8,40
		4,21	9,27
		12,05	8,02
		5,66	10,01
			8,11
