# Supplementary material for: Procollagen C-Proteinase Enhancer-1 (PCPE-1) deficiency in mice reduces liver fibrosis but not NASH progression
Source: PLoS One. 2022 Feb 11;17(2):e0263828. doi: 10.1371/journal.pone.0263828 (PMC8836302; doi:10.1371/journal.pone.0263828)
Supplement: S6 Raw dataset — Expression of PCOLCE (A) and PCOLCE2 (B) mRNA and PCPE-1 protein (C) in human liver samples (Fig 6). (PDF) [file pone.0263828.s012.pdf]

A

Control	NASH
1	0,89
0,83	0,13
0,64	0,25
0,73	1,11
0,94	0,38
1,72	
1,08	
0,31	

B

Control	NASH
0,89	0,18
0,13	0,03
0,25	0,08
1,11	1,06
0,38	0,48
2,24	
0,59	
0,17	

C

Control	NASH	HCV
1,39	2,22	3,69
2,20	3,04	3,33
1,15	8,30	2,68
1,52	3,60	3,55
