# Supplementary material for: Procollagen C-Proteinase Enhancer-1 (PCPE-1) deficiency in mice reduces liver fibrosis but not NASH progression
Source: PLoS One. 2022 Feb 11;17(2):e0263828. doi: 10.1371/journal.pone.0263828 (PMC8836302; doi:10.1371/journal.pone.0263828)
Supplement: S7 Raw dataset — (PDF) [file pone.0263828.s013.pdf]

**WT** *Pcolce*	**WT** *Pcolce2*	**WT** *Bmp1*	*Pcolce*$^{-/-}$ *Pcolce*	*Pcolce*$^{-/-}$ *Pcolce2*	*Pcolce*$^{-/-}$ *Bmp1*
0,70	1,43	0,91	0	0,97	1,10
1	1,03	1	0	0,95	1,08
0,77	1,20	1,04	0	0,65	1,02
0,87	1,73	0,99	0	0,62	1,17
1,55	0,97	1,24	0	0,55	1,11
1,082	0,90	1,08	0	1	0,93
1,12	1,27	1,12	0	0,84	0,89
0,98	1,07	1,12	0	0,77	1,43
1,056	0,56	1,05	0	0,97	0,87
			0	1,11	1,22
			0	0,96	1,21
			0		0,92
					1,15
					1,16
