# Supplementary material for: Procollagen C-Proteinase Enhancer-1 (PCPE-1) deficiency in mice reduces liver fibrosis but not NASH progression
Source: PLoS One. 2022 Feb 11;17(2):e0263828. doi: 10.1371/journal.pone.0263828 (PMC8836302; doi:10.1371/journal.pone.0263828)
Supplement: S8 Raw dataset — Body (A) and liver (B) weight, liver TG (C), ALT (D) and AST (E) levels in WT and Pcolce-/- female mice under A04 or CDA HFD after 8 weeks (S2 Fig). (PDF) [file pone.0263828.s014.pdf]

A

Time (w)	WT A04										
0	20,1	20,6	21,1	20	21,1	20,2	15,9	19,26	18,08	17,35	20,4
1	21,2	20,9	20,8	21	21,9	20,5	16,1	20,25	19,48	18,13	21,64
2	21,4	21	22,3	20,8	21,7	21,39	18,64	21,45	19,8	17,73	22,48
3	21,8	20,8	23,3	22,03	22,53	23,26	19,45	21,43	21,27	18,39	23,53
4	22,24	21,98	22,58	24,19	24,37	24,45	19,25	22,84	21,78	20,61	25,01
5	22,91	22,68	24,13	22,47	24,96	24,69	20,02	22,47	22,85	21,72	25,22
6	22,52	22,84	26,3	22,95	25,69	27,94	19,71	24,06	22,78	22,43	25,89
7	23,49	23,28	24,73	23,72	24,07	27,24	20,69	23,93	23,89	21	26,39
8	23,33	24,16	26,04	23,99	25,01	29,9	20,6	23,3	23,37	21,96	26,79

Time (w)	*Pcolce*$^{-/-}$ A04				
0	20,37	19,04	19,66	19	19,44
1	20,48	19,87	20,92	20,52	19,78
2	20,97	20,62	20,52	21,08	20,36
3	21,3	21,05	21,02	20,27	20,41
4	22,28	21,38	22,06	22,52	20,67
5	23,18	21,59	23,11	21,16	21,44
6	24,45	22,05	23,73	22,57	23,03
7	24,23	21,89	24,28	23,67	22,25
8	25,76	23,05	24,27	22,98	23,36

Time (w)	WT CDA HFD														
0	20,8	20,9	19,9	19,8	18,9	16,9	17,15	17,72	20,64	19,93	20,1	19,9	19,31	18,79	17,69
1	22,8	21,1	20,6	19,9	19,7	18	18,15	19	19,48	20,43	19,64	19,35	19,77	19,52	17,8
2	21,4	21,5	20,54	21,1	20,4	18,4	18,88	19,35	18,62	21,53	20,28	19,06	20,71	20,2	18,68
3	21,6	21,7	20,6	21,2	20,19	19,25	19,61	20,72	18,66	22,53	20,63	18,56	20,7	20,83	19,57
4	21,58	22,48	21,03	21,94	20,98	20,11	20,37	20,19	18,5	22,65	22	19,3	21,69	20,68	19,89
5	22,2	23,21	21,79	21,88	21,11	19,9	20,69	20,35	19,03	22,82	23,04	19,44	22,58	21,47	20,61
6	22,77	24,19	21,92	22,57	21,3	20,61	20,9	20,73	19,54	24,48	23,15	19,73	22,69	21,59	20,49
7	23,8	24,34	22,12	23,32	21,64	21,58	21,7	21,2	19,56	24,69	22,92	20,22	22,78	22,05	21
8	24	24,69	22,58	24,68	22,45	21,07	22,38	21,78	20,22	24,75	24,38	21,03	23,35	23,25	21,96

Time (w)	*Pcolce*$^{-/-}$ CDA HFD								
0	19,39	19,06	18,51	16,91	16,62	19,63	18,7	18,69	20,8
1	19,52	21,12	20,31	18,31	19,01	18,75	19,48	19,45	21,9
2	19,78	21,14	19,96	18,88	18,88	19,52	19,79	20,37	20,8
3	21,18	21,74	20,91	20,13	18,82	19,13	20,56	21,91	21,8
4	22,25	22,12	21,81	20,28	18,76	18,67	20,97	22,44	20,55
5	21,68	22,4	21,52	20,23	20,07	18,85	22,25	22,61	20,69
6	22,79	22,72	22,33	20,78	20,01	19,09	21,76	21,78	21,55
7	22,67	23,64	22,56	21,1	20,87	19,04	21,92	22,15	21,81
8	24	23,61	23,19	21,3	21,07	21,3	24,1	23,3	22,7

B

WT A04										
0,97	1,08	0,85	0,91	1,16	1,10	0,86	1,05	1,14	1,03	1,17

Pcolce$^{-/-}$ A04				
1,2377	1,0172	1,0728	0,9393	1,0119

WT CDA HFD														
1,63	1,71	1,46	2,22	1,52	1,57	1,68	1,51	0,95	1,78	1,67	1,06	1,57	1,73	1,55

Pcolce$^{-/-}$ CDA HFD								
1,76	1,59	1,64	1,39	1,58	1,31	1,85	1,66	1,56

C

WT A04									
27,1	41,3	14,8	25,8	37,2	36,2	27,7	20,9	23,9	31

Pcolce$^{-/-}$ A04				
45,8	22,6	24,9	20,9	25,5

WT CDA HFD														
210,1	251,2	198,9	160,6	196,9	171,5	216,1	201,4	222,9	178,2	216,3	212,1	200,6	151,8	229,9

Pcolce$^{-/-}$ CDA HFD								
172,3	221,5	181,4	211	180,5	149	246,2	201,3	221,7

D

WT A04									
22	20	16	22	23	19	21	92	36	27

Pcolce$^{-/-}$ A04	
25	22

WT CDA HFD										
228	203	193	357	330	327	202	214	243	260	196

Pcolce$^{-/-}$ CDA HFD					
466	336	189	314	302	248

E

WT A04									
96	67	64	66	65	59	76	180	146	95

Pcolce$^{-/-}$ A04	
85	98

WT CDA HFD										
187	194	214	391	357	382	246	271	671	251	345

Pcolce$^{-/-}$ CDA HFD					
503	413	226	356	328	286
