# Supplementary material for: Procollagen C-Proteinase Enhancer-1 (PCPE-1) deficiency in mice reduces liver fibrosis but not NASH progression
Source: PLoS One. 2022 Feb 11;17(2):e0263828. doi: 10.1371/journal.pone.0263828 (PMC8836302; doi:10.1371/journal.pone.0263828)
Supplement: S9 Raw dataset — NAS score (A), liver steatosis (B) and inflammation (C) in WT and Pcolce-/- female mice under A04 or CDA-HFD after 8 weeks (S3 Fig). (PDF) [file pone.0263828.s015.pdf]

A

WT A04	*Pcolce*$^{-/-}$ A04	WT CDA HFD	*Pcolce*$^{-/-}$ CDA HFD
0	1	6	5
0	1	5	5
2	1	6	5
1	1	4	6
2	1	5	6
1		6	6
1		6	6
0		4	5
0		6	
1		5	
0		5	
		6	
		5	
		5	

B

	0	1	2	3
**WT A04**	55	36	9	0
***Pcolce*$^{-/-}$ A04**	0	100	0	0
**WT CDA HFD**	0	0	0	100
***Pcolce*$^{-/-}$ CDA HFD**	0	0	0	100

C

	0	1	2	3
**WT A04**	73	18	9	0
***Pcolce*$^{-/-}$ A04**	60	40	0	0
**WT CDA HFD**	0	14	43	43
***Pcolce*$^{-/-}$ CDA HFD**	0	0	50	50
