# Supplementary material for: Procollagen C-Proteinase Enhancer-1 (PCPE-1) deficiency in mice reduces liver fibrosis but not NASH progression
Source: PLoS One. 2022 Feb 11;17(2):e0263828. doi: 10.1371/journal.pone.0263828 (PMC8836302; doi:10.1371/journal.pone.0263828)
Supplement: S10 Raw dataset — Liver fibrosis score (A), total collagen (B) and insoluble collagen (C) in WT and Pcolce-/- female mice under A04 or CDA-HFD after 8 weeks (S4 Fig). (PDF) [file pone.0263828.s016.pdf]

A

	0	1	2	3	4
**WT A04**	45	55	0	0	0
***Pcolce*$^{-/-}$ A04**	100	0	0	0	0
**WT CDA HFD**	0	43	43	14	0
***Pcolce*$^{-/-}$ CDA HFD**	0	50	50	0	0

B

WT A04	*Pcolce*$^{-/-}$ A04	WT CDA HFD	*Pcolce*$^{-/-}$ CDA HFD
2,76	4,55	9,97	4,32
7,47	3,69	3,98	4,24
5,71	4,6	8,37	4,3
		9,02	
		7,54	
		7,17	
		8,83	
		7,48	
		10,24	

C

WT A04	*Pcolce*$^{-/-}$ A04	WT CDA HFD	*Pcolce*$^{-/-}$ CDA HFD
7,09	3,49	4,19	6,63
9,07	3,61	11,1	6,97
7,5	7,18	9,29	5,64
4,66	1,23	12,92	3,07
2,47	5,04	8,04	6,86
7,8		4,69	2,28
10,5		9,36	5,4
5,28		12,49	1,8
4,07		8,48	6,15
3,85		9,26	
2,9		5,37	
		7,59	
		6,11	
		11,3	
